# A Quasi-Domesticate Relic Hybrid Population of *Saccharomyces cerevisiae* × *S. paradoxus* Adapted to Olive Brine

**DOI:** 10.3389/fgene.2019.00449

**Published:** 2019-05-29

**Authors:** Ana Pontes, Neža Čadež, Paula Gonçalves, José Paulo Sampaio

**Affiliations:** ^1^UCIBIO-REQUIMTE, Departamento de Ciências da Vida, Faculdade de Ciências e Tecnologia, Universidade Nova de Lisboa, Caparica, Portugal; ^2^Biotechnical Faculty, University of Ljubljana, Ljubljana, Slovenia

**Keywords:** yeast, *Saccharomyces cerevisiae*, hybridization, microbe population genomics, microbiology of olive brine

## Abstract

The adaptation of the yeast *Saccharomyces cerevisiae* to man-made environments for the fermentation of foodstuffs and beverages illustrates the scientific, social, and economic relevance of microbe domestication. Here we address a yet unexplored aspect of *S. cerevisiae* domestication, that of the emergence of lineages harboring some domestication signatures but that do not fit completely in the archetype of a domesticated yeast, by studying *S. cerevisiae* strains associated with processed olives, namely table olives, olive brine, olive oil, and alpechin. We confirmed earlier observations that reported that the Olives population results from a hybridization between *S. cerevisiae* and *S. paradoxus.* We concluded that the olive hybrids form a monophyletic lineage and that the *S. cerevisiae* progenitor belonged to the wine population of this species. We propose that homoploid hybridization gave rise to a diploid hybrid genome, which subsequently underwent the loss of most of the *S. paradoxus* sub-genome. Such a massive loss of heterozygosity was probably driven by adaptation to the new niche. We observed that olive strains are more fit to grow and survive in olive brine than control *S. cerevisiae* wine strains and that they appear to be adapted to cope with the presence of NaCl in olive brine through expansion of copy number of *ENA* genes. We also investigated whether the *S. paradoxus* HXT alleles retained by the Olives population were likely to contribute to the observed superior ability of these strains to consume sugars in brine. Our experiments indicate that sugar consumption profiles in the presence of NaCl are different between members of the Olives and Wine populations and only when cells are cultivated in nutritional conditions that support adaptation of their proteome to the high salt environment, which suggests that the observed differences are due to a better overall fitness of olives strains in the presence of high NaCl concentrations. Although relic olive hybrids exhibit several characteristics of a domesticated lineage, tangible benefits to humans cannot be associated with their phenotypes. These strains can be seen as a case of adaptation without positive or negative consequences to humans, that we define as a quasi-domestication.

## Introduction

The domestication of plants and animals was a major revolution in human history because it drove the emergence civilizations with the associated demographic and technological consequences that last until today (Diamond, 1997). In many instances domestication represents a dramatic case of adaptive divergence in response to human selection (Doebley et al., 2006; Ross-Ibarra et al., 2007). Domestication consists in the selective and controlled propagation an organism that genetically acquires modifications that not only distinguish it from its wild ancestors, but also make it more useful to humans (Diamond, 2002). Since archeological and biomolecular evidence indicates that fermented beverages reminiscent of rice wine were produced as far back as 9,000 years ago in China (McGovern, 2009) and the forebear of modern beer was consumed 8,000 years ago in Sumeria (Hornsey, 2003), the case of microbe domestication has a context and a time scale comparable to the much better understood aspects of plant and animal domestication. In fact, the mechanisms and consequences of artificial selection of microbes such as yeasts, carried out in most cases unconscientiously by innumerable generations of brewers, winemakers and other artisans, are starting to be understood (Almeida et al., 2015, 2017; Gallone et al., 2016; Gonçalves et al., 2016; Barbosa et al., 2018; Duan et al., 2018; Legras et al., 2018; Peter et al., 2018). Although the mechanisms that gave rise to the phenotypes of domesticated strains are currently the focus of intense scientific inquiry, the detailed comprehension of the multiple transformations that gave rise to domesticated yeast lineages is far from been achieved.

Besides the obvious cases of the emergence of wine, beer or sake variants, several other presumably domesticated lineages of *Saccharomyces cerevisiae* have also been recently revealed (Duan et al., 2018; Preiss et al., 2018). Moreover, the proximity to humans appears to have elicited the emergence of a new niche to which *S. cerevisiae* is adapting to, as the isolation of commensal (Angebault et al., 2013) and opportunistic (Enache-Angoulvant and Hennequin, 2005; Munoz et al., 2005) strains suggests. Recently we and others have explored the domestication space of *S. cerevisiae* (Almeida et al., 2015; Gallone et al., 2016; Gonçalves et al., 2016; Barbosa et al., 2018; Legras et al., 2018) and one of the main findings is that the domestication routes of this yeast are multiple and independent, and most remain poorly known. The picture that is gradually emerging depicts a complex population structure rich of different wild populations, most of them showing geographical partitioning, and numerous domesticated populations, each associated with a given fermented product and showing specific adaptations related to the type of fermentation in which they participate (Duan et al., 2018; Legras et al., 2018; Peter et al., 2018). This complex scenario is further complicated by the occurrence of admixture between certain populations that gives rise to mosaic genotypes (Tilakaratna and Bensasson, 2017) and to transitions from primary to secondary domestications (Barbosa et al., 2018). Here we address a yet unexplored aspect of *S. cerevisiae* domestication – that of the emergence of lineages harboring some domestication signatures but that do not fit completely in the definition of a domesticated yeast because artificial selection, even if unintentional, is not easy to accommodate with the emergence of a phenotype that provides identifiable benefits to humans. Specifically we survey a set of *S. cerevisiae* strains associated with the maturation of table olives, where they occur spontaneously without propagation from one batch to the other. Olive brine strains were first studied by Cromie et al. (2013) using restriction site-associated sequencing (RAD-seq), a reduced genome sequencing strategy. These authors detected a small group of *S. cerevisiae* strains isolated from European olives that clustered next to wine strains and were defined by a unique set of sequence variants not present in other populations. Subsequently, Strope et al. (2015), in a genomics survey of 100 *S. cerevisiae* strains, found that a restricted group of three strains that included YJM 1252 (=PYCC 6732 = CBS 3081), isolated from alpechin (olive mill wastes) in Spain had a considerable number of ORFs (>200) from *S. paradoxus*. The other two strains were YJM 1078 (NRRL YB-4348 = PYCC 8028) and YJM 248 (NRRL Y-12659 = CBS 2910 = PYCC 8034) isolated in Portugal in the 1950’s from human feces. In a more recent study involving the genome analyses of more than 1000 *S. cerevisiae* strains, Peter et al. (2018) identified 17 strains in a so-called alpechin clade sharing the already reported *S. paradoxus* contribution. Because the strains originating from the olive niche have never been studied separately but rather on comprehensive surveys that included hundreds or thousands of strains from other provenances, thus precluding their detailed analysis, we carried out an investigation on the ecological, physiological and genomic particularities of olive strains aiming at understanding their origins and specific adaptations within the global framework of yeast domestication. We found that olive strains are more fit to grow and survive in olive brine than control *S. cerevisiae* wine strains and that they appear to be adapted to cope with the presence of NaCl in olive brine. Moreover, the ecological range of these strains includes the processed olives niche but not olive trees or olives in natural conditions. We postulate that an ancient hybridization between a *S. cerevisiae* wine strain and *S. paradoxus*, provided the genetic diversity that allowed the adaptation to the new niche and that this process was accompanied by the adaptive loss of most the non-cerevisiae sub-genome.

## Materials and Methods

### Yeast Isolation, Identification, and Crosses

The isolation of *Saccharomyces* strains was conducted using a selective enrichment protocol previously described (Sampaio and Gonçalves, 2008). For the strains isolated in Slovenia, samples were directly used for yeast isolation without enrichment. Preliminary species-level identifications were performed by sequencing the D1/D2 region of the 26S rDNA. Crosses involved ascospore micro-manipulation. Positive crosses between two parental strains were confirmed by sequencing of *GAL1* and confirmation of the expected heterozygous sites. For each cross, interspecific spore viability was determined by examining 200 ascospores.

### Olive Brine Medium

Olives of the Oblica variety (approx. 200 g) collected in Évora, Portugal were used to prepare olive brine (200ml H_2_O, 8% NaCl w/v) during 3 months at 17°C. After this period, the brine was sterilized by filtration and kept at 4°C. This brine was analyzed by HPLC to identify and quantify the sugars and sugar-like compounds present. Sugar concentrations were determined using a carbohydrate analysis column (250 mm × 4 mm + Aminotrap, Dionex Carbopac PA10; DIONEX ICS3000). The column was kept at 25°C and 0.1M NaOH was used as the mobile phase at 1 ml min^-1^. For the phenolic compounds the concentrations (%) were determined using a Waters Novapack C18 15 mm column (DIONEX ICS3000). The column was kept at 30°C and 2% methanol was used as the mobile phase at 0.5 ml min^-1^.

### Absolute Fitness in Olive Brine

Two experiments were done independently for the group of strains selected. Pre-cultures (20 ml in 50 ml flasks) grown for 24 h in YNB (Yeast Nitrogen Base, Difco) supplemented with 1% (w/v) glucose (incubation at 25°C) were used to inoculate approximately 1 × 10^5^ cells/ml in a volume of 2 ml of olive brine in 2 ml micro-centrifuge tubes. Cells were grown in batch cultures for 70 days at 17°C without shaking, and cell viability was estimated by performing regular plate counts after preliminary counts in a hemocytometer. Statistical significance was tested using an unpaired *t*-test with Welch’s correction, implemented in GraphPad Prism v5 (*p*-value cut-off <0.01). At the end of the experiment, the supernatants of two cultures from the Olives population and two cultures from the Wine population were randomly chosen for HPLC analysis to determine the residual concentrations of sugars.

### Growth Rates in NaCl

Strains were pre-grown overnight in liquid YPD medium [yeast extract 1% (w/v), peptone 2% (w/v), D-glucose 2% (w/v), at 25°C and were subsequently transferred to fresh medium (20 ml YPD or YPD supplemented with NaCl 6% or 8% w/v) and incubated with orbital shaking (180 r.p.m.) at 30°C in 50 ml flasks]. The initial OD_640nm_ was 0.1 – 0.2. Growth rates were calculated in the exponential phase using OD_640nm_ measurements.

### Sugar Consumption in Phosphate Buffer

Strains were pre-grown overnight at 25°C in YNB medium supplemented with 1% (w/v) glucose. Cells were then transferred to phosphate buffer at pH 5.8 (30 ml in 50 ml flasks) supplemented with 0.6% (w/v) glucose 0.1% (w/v), fructose, and 8% (w/v) NaCl, to mimic the conditions in olive brine and incubated at 20°C. A similar experiment was also conducted in the absence of NaCl. Sugar consumption was monitored for 10 days by HPLC. Extracellular concentrations of fructose and glucose were determined using a carbohydrate analysis column (300 mm × 7 mm, Thermo HyperREZ XP Carbohydrate Ca++; KNAUER Smartline) and a differential refractometer. The column was kept at 85°C and H_2_O was used as a mobile phase at 0.6 ml min^-1^.

### Genome Sequencing, Read Alignment, and Genotype Calling

DNA was extracted from overnight grown cultures of monosporic or single-cell derivatives and paired-end Illumina MiSeq 250 bp genomic reads were obtained after sequencing for 500 cycles. Genomic data for other strains was obtained from the NCBI-SRA archive and from the *Saccharomyces* Genome Resequencing Project v2 (SGRP2) (Bergström et al., 2014). When only finished genome sequences were available in public databases (NCBI), the corresponding error-free Illumina reads were simulated using dwgsim^1^. Reads for each isolate were mapped to the *S. cerevisiae* reference genome (UCSC version sacCer3) using SMALT v0.7.5 aligner^2^. The reference Index was built with a word length of 13 and a sampling step size of 2 (-k 13 -s 2). An intensive search for alignments (-x) was performed during the mapping step with the random assignment of ambiguous alignments switched off (-r -1) and the base quality threshold for the look-up of the hash index set to 10 (-q 10). With these settings, SMALT v0.7.5 only reports the best unique gapped alignment for each read. For the paired-end data the insert size distribution was inferred with the “sample” command of SMALT prior to mapping. Conversion of SAM format to BAM, sorting, indexing, several mapping statistics, and consensus genotype calling were performed using the tools available in the SAMtools package v1.18 (Li et al., 2009) and as described previously (Almeida et al., 2014). Multiple sequence alignments for each reference chromosome were generated from the resulting fasta files. For downstream analysis, all bases with Phred quality score below Q40 (equivalent to a 99.99% base call accuracy) or ambiguous base calls were converted to “N.” For obtaining the *S. cerevisiae* and *S. paradoxus* sub-genomes of the hybrid strains, reads for each strain were mapped to an extended *Saccharomyces* spp. reference with assembled sequences from the genomes for *S. cerevisiae* (UCSC version sacCer3), *S. paradoxus*, *S. mikatae*, *S. kudriavzevii*, *S. uvarum* (Scannell et al., 2011), and *S. arboricolus* (Liti et al., 2013).

### Phylogenetic Inference and Divergence Across the Genome

Chromosomal single nucleotide polymorphisms (SNPs) were extracted from multiple sequence alignments only if the evaluated site was represented by unambiguous high confidence alleles in at least 85% of the strains. SNPs were then concatenated to generate a whole-genome SNP alignment. The phylogeny was inferred using maximum likelihood as implemented in IQ-TREE v 1.6.7 (Nguyen et al., 2015) using an empirically determined substitution model, SH-like approximate likelihood ration test (1000 replications) (Guindon et al., 2010), and rooted with *S. paradoxus*. The phylogeny was visualized using ITOL, version 3.0 (Letunic and Bork, 2016). Whole-genome levels of divergence were estimated using Variscan v2.0 (Hutter et al., 2006). Divergence was calculated for each mapped strain in comparison with the reference genome of *S. cerevisiae* using RunMode 21. The results were processed using a 10 kb sliding window with 10 kb step increments.

### Screening for Non-*S. cerevisiae* Genes

Evidence of the presence of genes from other *Saccharomyces* species was investigated by mapping the reads to a combined reference that includes the annotated coding sequences of *S. arboricola*, *S. cerevisiae*, *S kudriavzevii*, *S. mikatae*, *S. paradoxus*, and *S. uvarum* (Scannell et al., 2011; Liti et al., 2013). Reads were mapped to this combined reference using BWA V0.6.2 (Li and Durbin, 2009) with default parameters, but setting the quality threshold to 10 (-q 10). SAMtools V1.1852 (Li et al., 2009) was then used for manipulation of the resulting BAM files. Only ORFS with orthologs unambiguously annotated in all the species were analyzed. An ORF was considered to have a foreign origin to *S. cerevisiae* if its coverage was higher at least one-fourth of the median whole genome coverage for a given strain. The ORF coverage was defined as the product of the total number of mapped reads to a given ORF by the read length, dividing by the sum of all the ORFS length (considering only ORFS that have at least 25% of reads mapped to, when comparing to the orthologous ORF with the highest number of reads). This measure was taken to control spurious alignment counts. The coverage threshold allowed for some heterogeneity in the read counts and for the eventual presence of a foreign ORF together with the native *S. cerevisiae* ORF. For some of the *S. paradoxus* genes detected in the hybrid genomes, their assignment to this species was confirmed with phylogenetic analyses involving homologous sequences from other *Saccharomyces* species.

### Gene Ontology Analyses and Survey of Specific Genes and of Gene Copy Number Variation

Standard gene ontology (GO) term find was performed with the GO TERM FINDER tool v0.83, available at SGD, using a *p*-value cut-off of <0.01. We performed *de novo* genome assemblies using SPAdes v.3.11.1. Prior to assembly, reads were processed with Trimmomatic v.036 based on quality score threshold of 20 for windowed trimming, discarding reads less than 100 bp in length or harboring ambiguities. To retrieve genes of interest, a local BLAST database was set up for each genome. Copy number variation of the two *CUP1* genes (*CUP1-1* and *CUP1-2*) and the three *ENA* genes (*ENA1*, *ENA2*, and *ENA5*) was investigated using CNVNator (Abyzov et al., 2011) on mapped genomes and using *ACT1* as control. The query sequences were defined by the coordinates in the reference sequence of *S. cerevisiae* for the coding regions of the genes of interest. The results obtained were manually validated by checking the chromosomal context of the hits using UGENE (Okonechnikov et al., 2012) and by analyzing the copy number of the genes flaking the genes of interest.

### Data Availability

Genome sequence data have been deposited in the European Nucleotide Archive (ENA) database under the accession code PRJEB30431.

## Results

### Ecology and Phylogeny

Given earlier reports on the occurrence of *S. cerevisiae* in association with table olives (Arroyo-López et al., 2008; Cromie et al., 2013; Bonatsou et al., 2018) and with alpechin (Santa María, 1958), we asked if the original source of these yeasts was the olive tree itself. For this reason we conducted an isolation program employing samples of olive tree bark, leaves, fruits and soil underneath the trees, and a selective enrichment protocol for yeasts of the genus *Saccharomyces*. In parallel, processed products such as olive oil and olive brine from table olives were also investigated. In total 163 samples from olive trees were investigated, together with 53 samples from olive oil and 7 samples from olive brine. Although the number of samples collected from olive trees was much higher, the frequency of isolation of *Saccharomyces* spp. was very low (3.7%), and only six strains were collected. The frequency of isolation in olive oil was higher (7.6%), and yielded four strains but was still markedly lower than that of olive brine (85.7%, six strains). Therefore, in total 16 new strains of *Saccharomyces* spp. were isolated from the olive niche.

**FIGURE 1:**
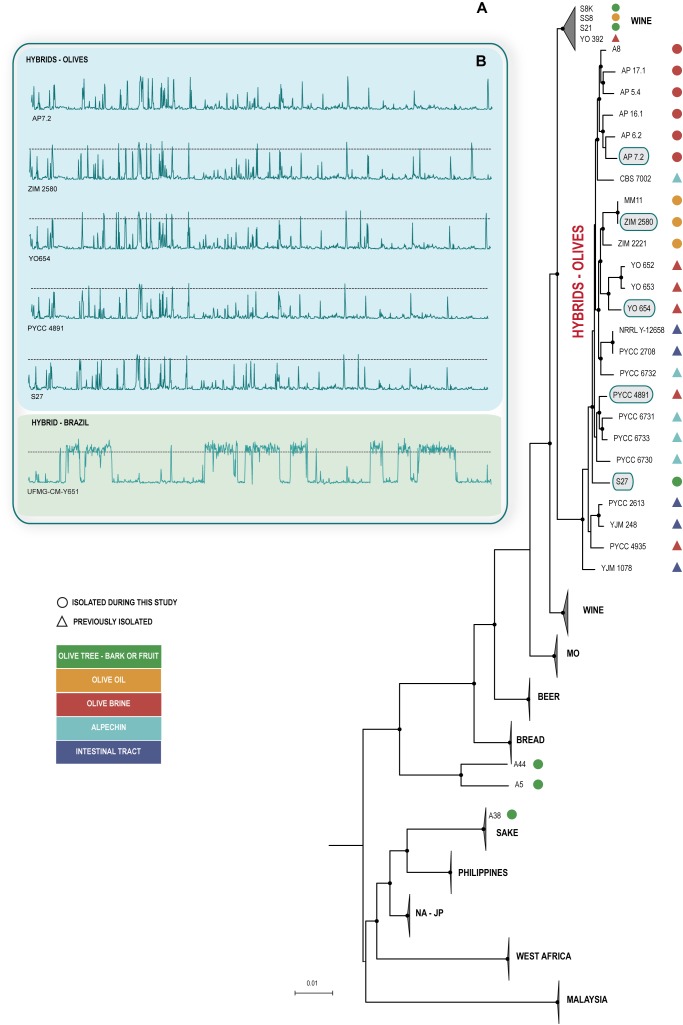
Hybrid olive strains form a monophyletic group and have a similar genomic organization. **(A)** Phylogenetic placement of hybrid olive strains among the known lineages of *Saccharomyces cerevisiae* (MO, Mediterranean oaks; NA-JP, North America – Japan). Whole-genome phylogenetic tree constructed after discarding *S. paradoxus* regions in all genomes. The phylogeny was inferred from of 93 sequences and 913590 SNPs using the Maximum Likelihood method as implemented in IQ-TREE with the TVM+F+G4 model of sequence evolution and was rooted with *S. paradoxus*. Branch lengths correspond to the expected number of substitutions per site and black dots in tree nodes depict bootstrap support values above 85% (1000 replicates). Strains isolated from the olive niche are distinguished based on the specific isolation source (see color codes). **(B)** Similar divergence plots of the genomes of selected hybrid strains (highlighted in the phylogeny) to the reference genome of *S. paradoxus* CBS 432. The dotted lines depict the 10% divergence threshold that represents the average divergence between *S. cerevisiae* and *S. paradoxus*. The substantially distinct divergence plot of a Brazilian *S. cerevisiae* × *S. paradoxus* hybrid strain (UFMG-CM-Y651) previously reported by us (Barbosa et al., 2016) is included for comparison.

Next, we obtained draft genome sequences of the new isolates in order to ascertain if they belonged to *S. cerevisiae* and, if so, to determine to which population they belonged to. As shown in Figure 1A, the new isolates were all identified as members of *S. cerevisiae*, thus showing that *S. paradoxus* was not isolated during our survey. Interestingly, the *S. cerevisiae* strains were found to belong to different populations. The six strains isolated directly from olive tree bark or ripe olives did not belong to the same population (Figure 1A and Supplementary Table S1). A single strain had substantial genomic contributions from *S. paradoxus* and, accordingly, was assigned to the Olives clade. Two strains belonged to the Wine clade, one strain to the Sake clade and two additional strains occupied an isolated position in the phylogeny and subsequent analyses showed that they had “mosaic” genomes with major contributions from the Wine and North American – Japan clades. With respect to the four strains isolated from olive oil, three of them had *S. paradoxus* contributions and belonged to the Olives clade. The remaining strain belonged to the Wine clade. For the strains isolated from olive brine, more homogeneous results were obtained and all of them were found to belong to the Olives clade (Figure 1A). Our phylogenetic analysis included also other strains that belong to the Olives clade and that had been isolated as far back as 1957 from olives, olive brine, alpechin, and from the gut or feces of humans and pigs (van Uden and Assis-Lopes, 1957; Santa María, 1958, 1962). It is noteworthy that the 25 *S. cerevisiae* × *S. paradoxus* hybrid strains isolated during this study or in previous studies formed a monophyllum even if the phylogeny of Figure 1A was prepared only with *S. cerevisiae* ORFs, thus avoiding the strong bias that would be introduced if *S. paradoxus* ORFs were considered. This suggests that all hybrid strains share the same *S. cerevisiae* ancestor irrespective of the geographical origin and particular substrate from which they were collected, a possibility also supported by the divergence plots depicting the *S. cerevisiae* and *S. paradoxus* blocks along the genome, that were similar for all strains of the Olives clade (Figure 1B). Moreover it appears the *S. cerevisiae* ancestor of the hybrids was a member of the wine population.

In conclusion, the results from our ecological survey do not support the hypothesis that the members of the Olives clade reside in the olive tree environment. Although the possibility that such strains are associated with olive trees cannot be entirely ruled out, it appears more likely that the ecological niche of this clade are processed olives and their products like olive oil, alpechin, which is the corresponding waste product, and table olives /olive brine. Also, the occurrence of hybrid strains in the intestinal tract is of notice. Besides the two strains (YJM 248 and YJM 1078) already reported in Strope et al. (2015) we found three additional strains from this source (PYCC 2613, PYCC 2708, and PYCC 8033).

### Fertility

Most strains (70%) of the Olives clade were sexually competent (Supplementary Table S1). Spore viability for two strains in this clade (PYCC 4935 and YO652) ranged 95.5 – 96% and a cross between them was also fertile (90% spore viability), thus suggesting that sexual recombination within the clade can occur. Also, a cross between YO 652 and the wine strain EXF 6719 (97% spore viability) had an ascospore fertility of 87%, thus indicating that sexual contact between the Olives and Wine populations appears not to be significantly hampered.

### Genomic Analysis

In order to characterize the genomic nature of the hybrids, we analyzed in detail 23 strains (Supplementary Dataset S1). We detected a total of 540 *S. paradoxus* ORFs and between 193 and 314 *S. paradoxus* ORFs per strain, with 103 ORFS being shared among all the strains. The *S. paradoxus* ORFs originated in the European population of this species (Supplementary Figure S2), thus suggesting that the hybridization event occurred in this continent. The co-existence of *S. cerevisiae* and *S. paradoxus* alleles for a given ORF was not frequent. In total 148 ORFs (27.4% of the total number of *S. paradoxus* ORFs) were found to occur in that configuration in at least one strain. One strain was devoid of ORFs represented in the genome by alleles belonging to the two species and 16 strains had only two to four ORFs with *S. cerevisiae* and *S. paradoxus* alleles. Together, these strains represent 74% of the total number of strains analyzed. Three strains had between 12 and 25 ORFs with *S. cerevisiae* and *S. paradoxus* alleles and another three strains had between 34 and 54 ORFs with *S. cerevisiae* and *S. paradoxus* alleles. The distribution of strains having more heterozygous ORFs did not show any association with the isolation substrate or with the phylogeny.

**FIGURE 2:**
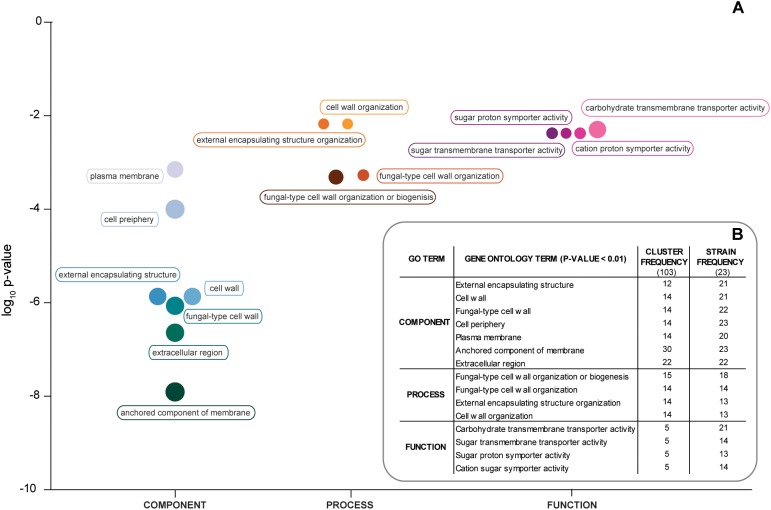
Gene ontology of *S. paradoxus* genes found in hybrid strains. **(A)** Gene ontology terms with *p*-value < 0.01 and organized under “Component,” Process,” and “Function” for the 103 *S. paradoxus* genes shared between the 23 hybrid genomes analyzed. The size of the circles is proportional to the number of genomes that contribute for that term. **(B)** Number of *S. paradoxus* genes and number of strains by GO (gene ontology) term.

Gene Ontology analysis of the 103 ORFs shared among all the strains revealed a significant enrichment in genes encoding for proteins of the fungal cell wall and plasma membrane, like *TIP1*, *HLR1*, *DAN1*, *FCY21*, and *STL1.* However, for several strains, when an individual analysis was performed, a significant result for an enrichment in hexose transporters was also observed (Figure 2 and Supplementary Table S2). Taken together, these results support the view that the hybrid strains have a similar core genomic composition, thus suggesting that they share a common (hybrid) ancestor and also that after hybridization have evolved adaptations to the processed olives niche.

Given that the hybrid strains descend from a *S. cerevisiae* wine strain (Figure 1A), we surveyed the hybrid genomes for the presence of typical domestication signatures of wine strains (Almeida et al., 2015; Barbosa et al., 2018). For regions A, B, and C, that encompass 39 genes potentially relevant for the winemaking process acquired by horizontal gene transfer from non- *Saccharomyces* species, at least one of these regions was present in 68.8% of the control group of 32 wine strains listed in Supplementary Table S1, whereas only 13% (3 out of 23) hybrid genomes shared the same characteristic. It thus appears that these regions are less prevalent in hybrid strains, either because their ancestor *S. cerevisiae* wine strain already lacked most of them and/or because there are not relevant in the olives niche and were therefore lost. With respect to the inactivation of aquaporin genes *AQY1* and *AQY2*, associated with the domestication of wine strains and with the adaptive loss of those water channels, a trait that increases fitness in sugar-rich environments (Will et al., 2010), no differences were found between the two groups and all strains had at least one aquaporin gene coding for a non-functional protein. We also investigated the variation of the number of copies of *CUP1*, a gene involved in resistance to copper toxicity in *S. cerevisiae*, especially in wine strains, due probably to their expose to copper sulfate used in vineyards (Fay et al., 2004; Strope et al., 2015). Copy number variation (CNV) of the two paralogs of *CUP1* among reference wine and wild (oak-associated) strains is shown in Supplementary Table S1. Whereas among wine strains CNV of *CUP-1* could exceed 30 (in two cases), the Mediterranean oak (MO) strains did not show an enrichment in the number of *CUP-1* copies. Some of the hybrid strains showed also elevated numbers of copies of *CUP-1*, with the two most enriched genomes having 33 and 35 copies. Statistically, wine and olives strains could not be distinguished in terms of presence and expansion of *CUP-1*.

### Absolute Fitness in Olive Brine

**FIGURE 3:**
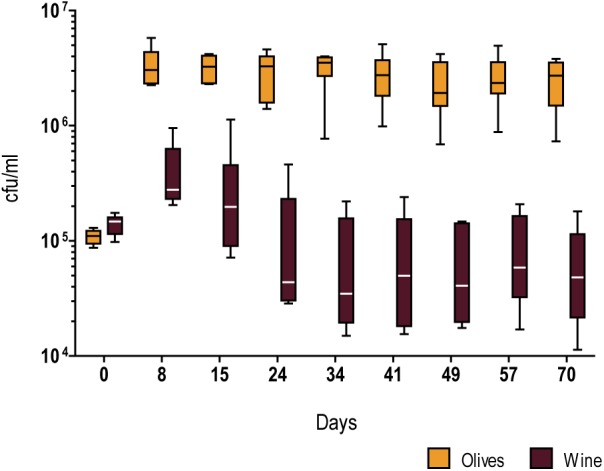
Whiskers plots of the relative fitness (growth and survival) in olive brine of representatives of the Olives (AP 5.4, AP 7.2, YO 654, ZIM 2580, PYCC 4891, and PYCC 6732) and Wine (AWRI 1631, Lalvin W15, PR, PYCC 4072, TUM V1, and Uvaferm VRB) populations of *S. cerevisiae*. The results are based on counts of colony forming units/ml of 6 strains from each group inoculated individually in two duplicate and independent experiments.

In order to investigate whether strains of the Olive clade were adapted to thrive in the processed olives niche, we estimated absolute fitness of a set of six strains isolated from olive brine, olive oil, and alpechin, and compared it to six *S. cerevisiae* strains from the wine population. We measured absolute fitness as the number of viable cells maintained in a long-term batch culture of table olive brine, here used as a proxy for a habitat to which the *S. cerevisiae* hybrids are adapted to (Figure 3, Supplementary Figure S1 and Supplementary Table S3). Employing freshly collected ripe olives, we prepared a brine containing 8% (w/v) NaCl (see section “Materials and Methods” for details) which was used to test each strain separately. The absolute fitness of the olive and wine strain cohorts was inferred by measuring viable cell numbers for 70 days in two independent experiments (Figure 3, Supplementary Figure S1 and Supplementary Table S3). Although strain fitness varies within both groups, variation is much more pronounced in the wine group. In spite of the within-group differences among strains, a clear difference is observable between fitness of the olive group and the wine group (*p* < 0.0001, unpaired *t*-test with Welch’s correction), the former being able both to attain higher cell numbers and to sustain viability throughout the duration of the experiment (70 days). On the contrary, wine strains tended to start losing viability already during the first month of incubation.

We reasoned that one possible cause for the difference in fitness between the two groups might be related to their ability to use the nutrients available in olive brine. Contrary to what is typical of the initial stages of wine fermentation, olive brine has low concentrations of sugars. To analyze this in more detail, we identified and quantified the sugars and sugar-related compounds present in olive brine and measured their consumption by two representatives of the wine and two representatives of the olive cohorts (Table 1). While strains belonging to the Olives clade virtually exhausted the glucose and fructose present initially in the brine, the representatives of the Wine clade consume only about half of the available sugars. Mannitol was left untouched in both cases.

**Table 1:** Sugar consumption in olive brine by strains of the Olives and Wine population of *S. cerevisiae*.

Time (days)	Population	Strain	pH	Glucose % (w/v)	Fructose % (w/v)	Mannitol % (w/v)
0			5.1	0.57	0.1	0.14
70	Olives	YO 654	5.0	0.0055	0.0023	0.13
		AP 7.2	4.9	0.0058	0.0039	0.14
	Wine	Lalvin W15	5.2	0.26	0.077	0.13
		TUM V1	5.2	0.38	0.083	0.14


*Initial and final (after 70 days) pH and sugar measurements are shown as averages for each strain from two independent experiments.*

This finding was intriguing because some wine strains were previously found to have an impairment in high affinity hexose transport (the type of transporters expressed under the low sugar concentrations measured in olive brine), a trait that was subsequently associated to certain variants of the HXT hexose transporter genes (Luyten et al., 2002). Numerous HXT genes are present in the genomes of the species of the genus *Saccharomyces*, encoding transporters with different affinities for their substrates. Since hexose transporter genes encoding the main high affinity transporters (HXT6/7) were among those “replaced” in the hybrids by their *S. paradoxus* counterparts (Supplementary Dataset S1), we asked if these substitutions might have contributed to improve high affinity hexose transport in the hybrids.

**FIGURE 4:**
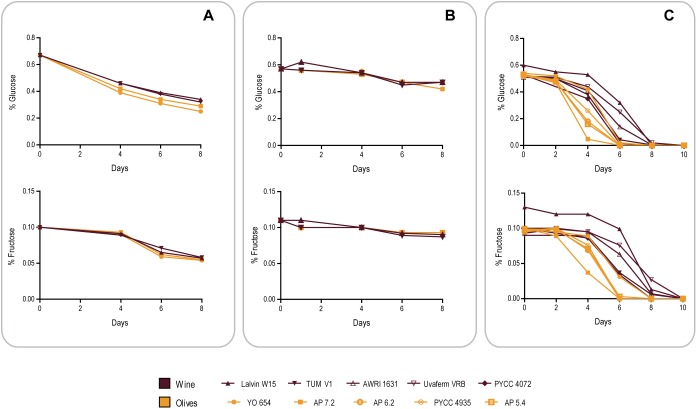
Comparison of glucose (initial concentration 0.6% w/v) and fructose (initial concentration 0.1% w/v) consumption by representatives of the Olives and Wine population of *S. cerevisiae* in different conditions. **(A)** Olive brine. **(B)** Phosphate buffer supplemented with 8% w/v NaCl. **(C)** Phosphate buffer supplemented with 8% w/v NaCl and 0.1% w/v yeast extract.

To assess this, we compared the ability of the same strains of the Wine and Olives clades used in the previous experiment (see Table 1) to consume the sugars present in olive brine in the course of the first 8 days after inoculation of brine (Figure 4A). Surprisingly, and although in this period olive strains grew on average in the brine fitness experiments shown in Figure 3 an order of magnitude more than wine strains, sugar consumption was very similar between wine and olive strains. Fructose consumption in particular was indistinguishable, while olive strains seemed to assimilate glucose slightly better, an observation that nevertheless does not explain the differences in growth in brine between the two sets of strains (Figure 3). A distinct experiment was subsequently performed in which brine was replaced by phosphate buffer supplemented with NaCl, glucose and fructose in concentrations identical to those found in brine. This time wine strains seemed to be slightly more proficient in fructose assimilation while in glucose no clear differences were observed (Figure 4B). Nevertheless, when this experiment was performed without NaCl, glucose and fructose were totally consumed after 2 days by wine and olives strains. Taken together these results suggest that no considerable differences in sugar uptake capacities exist between the two groups of strains that justify the better growth of olive strains. It seems therefore that the observed difference in growth is due to a better capacity to adapt to the harsh conditions of olive brine, of which high salt concentrations stand out, resulting in better growth for olive strains during the first 8 days while consuming the same amount of sugar in the same period as wine strains. The same experiment as shown in Figure 4B was subsequently performed but this time adding 0.1% yeast extract to the phosphate buffer and adding more strains to both cohorts to increase representativeness (Figure 4C). The sugar consumption profiles of both strain cohorts were different in these conditions, with olive strains exhausting the available sugars significantly faster (*p* < 0.05, unpaired *t*-test), which means that in the presence of the required nutrients olive strains are better equipped to adapt to the high salt medium. Interestingly, under these conditions wine strains were capable of consuming all the sugar available after 8–10 days while they only consumed about 50% of the available sugars during the fitness experiments in brine. This could mean that brine contains other inhibitors that affect the metabolism of wine strains more than that of olive strains, in addition to NaCl.

In summary, while *S. paradoxus* HXT genes may confer a slight advantage for glucose consumption in brine, this advantage does not explain the considerable difference in the ability of wine and olive strains to grow in brine. Instead, this difference seems to be derived from a better adaptation of olive strains to the particular conditions of brine of which the high NaCl concentration appears as a relevant factor.

### Adaptation to NaCl

**FIGURE 5:**
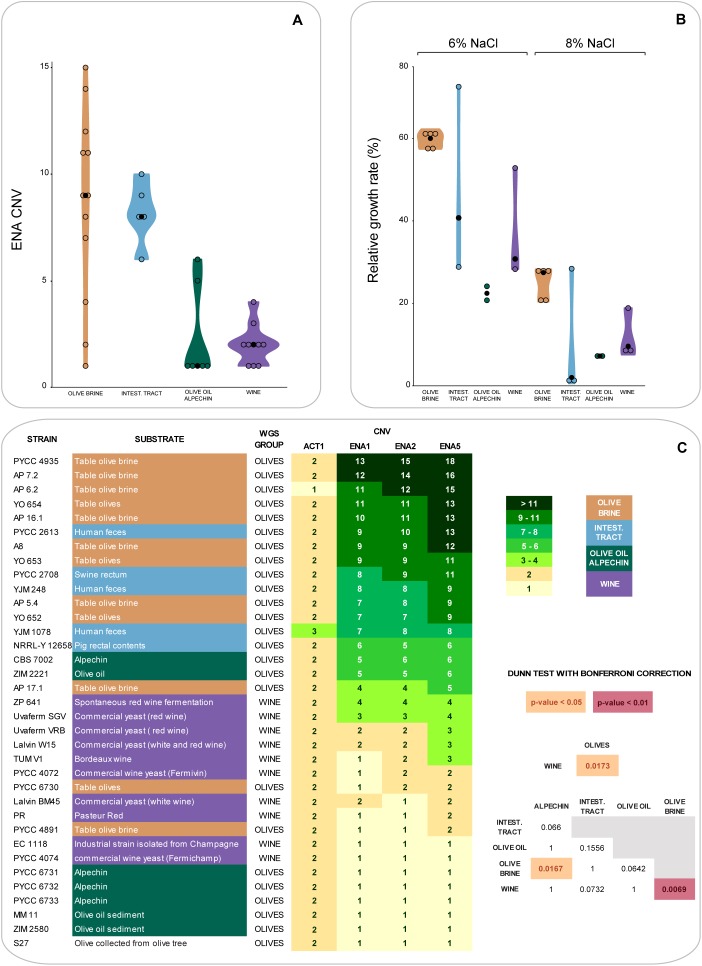
Copy number variation (CNV) of *ENA* genes and growth rates in the presence of NaCl of strains from the Olives and Wine populations. **(A)** Violin plots describing the number of *ENA* genes (*ENA1*, *ENA2* and *ENA5*, average value for each strain) among olive brine, intestinal tract, olive oil-alpechin, and wine strains (black circles indicate the median within each group). **(B)** Violin plots of relative growth rates in the presence of 6 and 8% (w/v) NaCl (reference: medium without NaCl) among olive brine, intestinal tract, olive oil-alpechin, and wine strains. **(C)** Numbers of *ENA* copies shown in tabular format for each strain. Darker green color shades correspond to increased numbers of gene copies. CNV of actin (*ACT1*) is indicated as reference. Statistical significant differences of CNV between groups of strains are highlighted.

The results of the experiments shown in Figure 3, 4 suggested that fitness and sugar consumption aptitude in brine might be related, at least partly, to salt resistance. To investigate this hypothesis, we started by determining the copy number of the three *ENA* genes found in the *S. cerevisiae* reference genome (*ENA1*, *ENA2*, and *ENA5*) in the hybrid strains of the Olives clade and compared their abundance using 10 representative strains of the Wine clade. The ENA proteins are sodium pumps that help the cells to cope with an excess of sodium ions in their environment (Ruiz and Ariño, 2007). *ENA* copy number variation is shown in Figure 5A,C. Interestingly, the highest *ENA* copy number (14–18 copies) was detected among strains isolated from olive brine (Figure 5C), although a marked dispersion in the number of *ENA* copies was observed in this group (Figure 5A). Overall, strains isolated from olive brine and the intestinal tract were more likely to have a higher number of *ENA* copies than strains isolated from olive oil, alpechin or wine (Figure 5A). It is possible that the strains found in the intestinal tract originate from the olive brine environment, having been subsequently ingested. This would explain their increased number of *ENA* copies. The differences between the number of *ENA* copies were found to be statistically significant between the Wine and Olive Brine populations (Figure 5C, *p* < 0.01, Dunn *post hoc* test with Bonferroni correction). The comparison of *ENA* copy numbers was also significantly distinct when all hybrid strains from the olives niche were compared with wine strains and for the comparison between the olive brine and alpechin groups (*p* < 0.05).

To investigate to which extent *ENA* gene copy numbers determined fitness of the strains under study in the presence of salt, the ability of the various strains to grow in the presence of 6% and 8% NaCl was also tested (Figure 5B). There was, as expected, a correlation between *ENA* gene copy numbers and the ability to grow in the presence of NaCl, but this correlation was not complete. For example, while all strains isolated from olive brine performed well in the growth tests even if they had only moderately high *ENA* copy numbers (e.g., AP 17.1; 4–5 copies), strains associated with the intestinal tract behaved heterogeneously, varying between an excellent performance (PYCC 2613; 9–13 copies) and a very poor performance (e.g., YJM 1078; 7–8 copies).

## Discussion

Here we analyzed in detail a *S. cerevisiae* × *S. paradoxus* hybrid lineage associated with a distinctive artificial environment, that of processed olives. Even when only *S. cerevisiae* ORFs are considered, these hybrid strains form a distinct and exclusive monophyletic lineage among those already known for *S. cerevisiae*. This suggests that the olive hybrids are genetically isolated from the other *S. cerevisiae* populations and that it is likely that they descend from a single ancestral hybridization event. A set of other additional features also suggests that olive hybrids constitute a “natural” population in an evolutionary and ecological sense. First, sexual recombination appears to be possible within members of this population and secondly the olive hybrids have a considerable dissemination both in space and in time, since in this study we analyzed representatives from the Iberian Peninsula (Portugal and Spain), Southeast Europe (Slovenia and Croatia), and also strains from the United States. Moreover, these strains were collected during a period of time that spans six decades (1957–2018). The Olives population exhibits a particular ecological preference to environments having in common the presence of processed olives or their products, but not the olive tree itself. Therefore it appears that the origin of this population is linked to human activities and to artificial substrates they create. Although some strains were found associated to the intestinal tract, these strains exhibit the characteristic expansion of the *ENA* gene copy number typical of olive brine strains, suggesting that they may have been ingested together with cured olives. The occurrence of *S. cerevisiae* hybrids in the intestinal tract parallels other reports of association of *S. cerevisiae* with humans (Angebault et al., 2013; Strope et al., 2015) and warrants the need for investigating if these strains are better adapted to survive in the intestinal tract.

**FIGURE 6:**
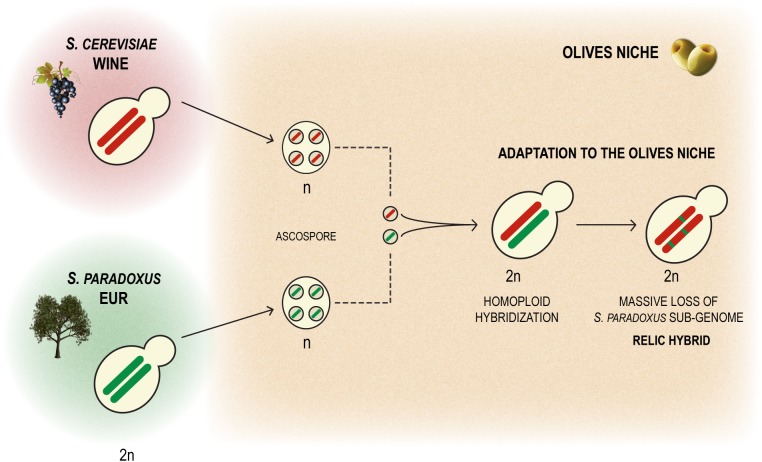
Model for the origin of relict *S. cerevisiae* hybrids as a consequence of homoploid hybridization between a *S. cerevisiae* wine strain and a *S. paradoxus* member of the European population, followed by adaptive LOH corresponding to a massive loss of the *S. paradoxus* sub-genome.

A striking feature of the genomes of the strains of the Olives population is the markedly unbalanced contribution of the two parental sub-genomes, *S. cerevisiae* being the clearly prevalent sub-genome given that *S. paradoxus* contributes only around 3.7%. A likely scenario for the origin of the hybrid that originated the Olives lineage is homoploid hybridization. This would have corresponded to the fusion of a *S. paradoxus* meiospore with a *S. cerevisiae* meiospore resulting in a “normal” diploid hybrid genome that subsequently underwent the adaptive loss of most of the *S. paradoxus* sub-genome (Figure 6). Therefore the observed genomic organization of the strains of the Olives lineage can be seen as reminiscent of a relic hybridization. This hybridization appears to have occurred in Europe since the *S. paradoxus* progenitor belongs to the recognized population of this species. We could determine that the *S. cerevisiae* progenitor belonged to the Wine population and that the hybrids still exhibit some of the domestication signatures of this population such as the loss of functional aquaporins, the expansion of *CUP-1* genes and the presence of region B, reminiscent of the presence of regions A, B, and C, typical in wine strains. It is noteworthy that these relic hybrids are capable of sexual reproduction, which would have facilitated the emergence of a population adapted to a newly colonized niche. The ecological barrier between the processed olives niche and the vineyards/winery environment, even if incipient would also have promoted, together with selection, the ecological specialization of the new genotypes. Therefore, the model we propose to explain the emergence of the Olives population is based on an original homoploid interspecies hybridization followed by a massive adaptive loss of heterozygosity (LOH) by replacement of most *S. paradoxus* alleles by their *S. cerevisiae* orthologs, combined with intra-population gene flow through sexual recombination and evolution of new ecological adaptations, with backcrossing with the *S. cerevisiae* parent probably playing a very limited role. Similar cases of apparent reduction of the non-*cerevisiae* sub-genome have also been reported for artificially generated hybrids involving *S. kudriavzevii* (Lopandic et al., 2016) and *S. uvarum* (Antunovics et al., 2005). Most importantly, LOH following hybridization, i. e. after a dramatic gain of genetic variation through interspecies hybridization, has been revealed as a major adaptation mechanism of populations when they invade new ecological niches (Smukowski Heil et al., 2017). Contrary to previous examples known exclusively from experimental evolution studies in the laboratory (e.g., Dunn et al., 2013; Smukowski Heil et al., 2017), the Olives population illustrates the fate of a relic hybridization in real conditions. It is also relevant to mention that although the fraction of *S. paradoxus* genome is relatively small, it is still much larger than instances of introgression of *S. paradoxus* in *S. cerevisiae* reported so far (Doniger et al., 2008; Barbosa et al., 2016), excluding the cases reported by Muller and McCusker (2009), in which information on strain origin was not given but that in fact correspond to the intestinal strains studied here. The relatively high number of homozygous *S. paradoxus* ORFs (342 out of 540) found in the Olives clade suggests that the genomic contribution of this species to adaptation to the processed olives environment is likely to involve a multiplicity of cellular processes. Gene ontology analysis of the set of *S. paradoxus* genes present in homozygosity in all hybrid strains examined here, suggested that cell wall function and hexose transport were likely among the cellular processes benefiting from the *S. paradoxus* genomic contribution. Because inefficient high affinity hexose transport was found to be associated with specific HXT alleles carried by some wine strains (Luyten et al., 2002), we investigated whether the *S. paradoxus* HXT alleles retained by the olives strains were likely to contribute to the observed superior ability of these strains to consume sugars in brine. However, our experiments indicate that sugar consumption profiles are different between members of the Olives and Wine populations solely in the presence of NaCl and only when the cells are cultivated in nutritional conditions that support adaptation of their proteome to the high salt environment. Assuming that the HXT transporters operating when cells are cultivated with or without a nitrogen source are in both instances the high affinity HXT6/7 transporters, these observations suggest that the differences perceived are due to a better overall fitness of olives strains in the presence of high NaCl concentrations, rather than to a better intrinsic ability of the *S. paradoxus* HXT6/7 versions to operate in the presence of salt. We observed that the strains isolated from olive brine had a tendency for having an increased number of copies of *ENA* genes, a feature known to increase tolerance to NaCl. However this tendency was not universal among olive brine strains and even strains with a lower number of *ENA* copies grew relatively well in the presence of NaCl, thus suggesting that other mechanisms might also be involved in the adaptation to NaCl of olive brine strains, as has been already documented (Posas et al., 2000; Dhar et al., 2011; Saito and Posas, 2012).

Interestingly, wine strains were able to exhaust glucose and fructose in the medium containing high NaCl concentrations while they failed to do so in brine even after 70 days, suggesting that inhibiting components other than NaCl are affecting the performance of wine strains in brine. Also, according to our results, these inhibitory components were not the phenolic compounds likely present in brine, since we failed to detect differences in the sensitivity of wine and olives strains (4 strains from each group) to oleuropein (3% w/v in YPD medium, pH 5), and ferulic acid (2% w/v in YPD medium, pH 4.5).

The emergence of the relic olive hybrids from an already domesticated lineage (wine yeasts) in the artificial environment of processed olives can be seen as another instance of yeast domestication, or even a case of secondary domestication *sensu* Barbosa et al. (2018). However, contrary to wine and beer domestication, where genomic and phenotypic changes can be linked to characteristics of these beverages valued and improved over time by humans, in the present case the beneficial role of the relic hybrids has not been clearly demonstrated in olive brine fermentations and therefore their origin and prevalence in the processed olives niche can be viewed as inconsequential to humans. One illustrative example of a tangible consequence of domestication is the inactivation of *PAD1* and *FDC1* genes in beer yeasts which overcomes the phenolic off flavor defect (Gallone et al., 2016; Gonçalves et al., 2016). The phenolic aroma, due the formation of 4-vinyl guaicol, is negatively valued in most in beers but not in wine where it can be even considered as desirable. The consequence of artificial selection is that beer yeasts differ from wine and wild strains in having acquired inactivating mutations in *PAD1* and *FDC1*. Therefore, if domestication is viewed as the controlled bred of an organism that becomes genetically distinct from its wild relatives in ways making it more useful to humans (Diamond, 2002), relic olive hybrids can be seen as a case of adaptation to the human environment but without the emergence of traits that we can readily recognize as useful. In order to reflect this distinct stage of “incomplete” domestication we define these changes as a quasi-domestication event.

## Author Contributions

AP, JS, and PG conceived the study, analyzed the data, and wrote the manuscript. AP and NČ performed the experiments. AP prepared the figures.

## Conflict of Interest Statement

The authors declare that the research was conducted in the absence of any commercial or financial relationships that could be construed as a potential conflict of interest.

## Footnotes

1 https://sourceforge.net/projects/dnaa/files/dwgsim/

2 http://www.sanger.ac.uk/resources/software/smalt/

## Supplementary Material

Figure S1 Growth and survival in olive brine of six strains of the Olives population and six strains of the Wine population. Two independent experiments were performed for each strain.

Figure S2 The *S. paradoxus* sub-genome of hybrid strains originates in the European population of *S. paradoxus*. Phylogenetic tree constructed with the *S. paradoxus* fraction of the hybrid genomes and correspondent regions from representatives of the European, American, and Far Eastern population of *S. paradoxus*. The phylogeny was inferred from 47 sequences and 22052 SNPs using the Neighbor Joining method and the P-distance model of sequence evolution. Branch lengths correspond to the expected number of substitutions per site and black dots in tree nodes depict bootstrap support values above 90% (1000 replicates).

Table S1 Strains and genomes used in this study and relevant information pertaining to them.

Table S2 Gene ontology (GO) analysis of the *S. paradoxus* sub-genome of relict hybrid strains.

Table S3 Cfu/ml counts per strain and per replicate for the growth and survival in olive brine experiment shown in Figure 2.

DATASET S1 Gene content of the *S. paradoxus* sub-genome of relict hybrid strains.
