# Supplementary material for: A Quasi-Domesticate Relic Hybrid Population of Saccharomyces cerevisiae × S. paradoxus Adapted to Olive Brine
Source: Front Genet. 2019 May 29;10:449. doi: 10.3389/fgene.2019.00449 (PMC6548830; doi:10.3389/fgene.2019.00449)
Supplement: Figure S1 — Growth and survival in olive brine of six strains of the Olives population and six strains of the Wine population. Two independent experiments were performed for each strain. [file Image_1.pdf]

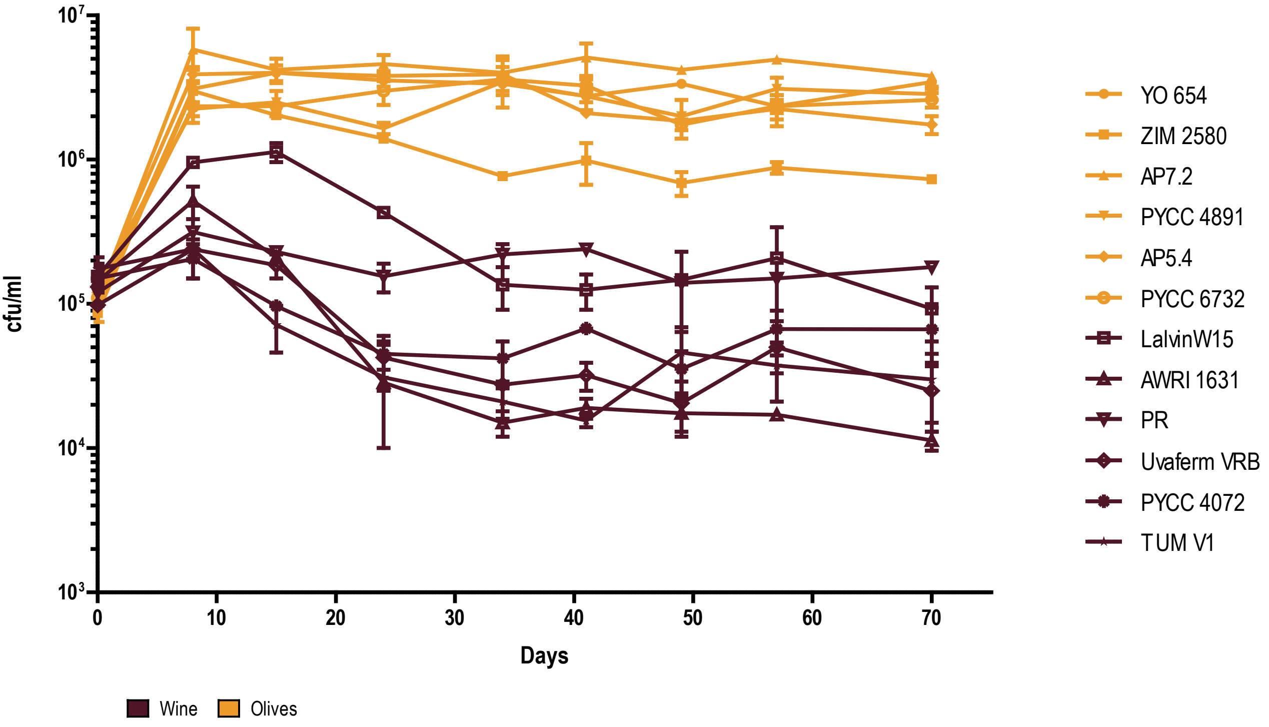

SUPPLEMENTARY FIGURE S1. Two replicate assays of growth and survival in olive brine of representatives of the Olives and Wine populations of *S. cerevisiae*.
