# Supplementary material for: A Quasi-Domesticate Relic Hybrid Population of Saccharomyces cerevisiae × S. paradoxus Adapted to Olive Brine
Source: Front Genet. 2019 May 29;10:449. doi: 10.3389/fgene.2019.00449 (PMC6548830; doi:10.3389/fgene.2019.00449)
Supplement: Figure S2 — The S. paradoxus sub-genome of hybrid strains originates in the European population of S. paradoxus. Phylogenetic tree constructed with the S. paradoxus fraction of the hybrid genomes and correspondent regions from representatives of the European, American, and Far Eastern population of S. paradoxus. The phylogeny was inferred from 47 sequences and 22052 SNPs using the Neighbor Joining method and the P-distance model of sequence evolution. Branch lengths correspond to the expected number of substitutions per site and black dots in tree nodes depict bootstrap support values above 90% (1000 replicates). [file Image_2.pdf]

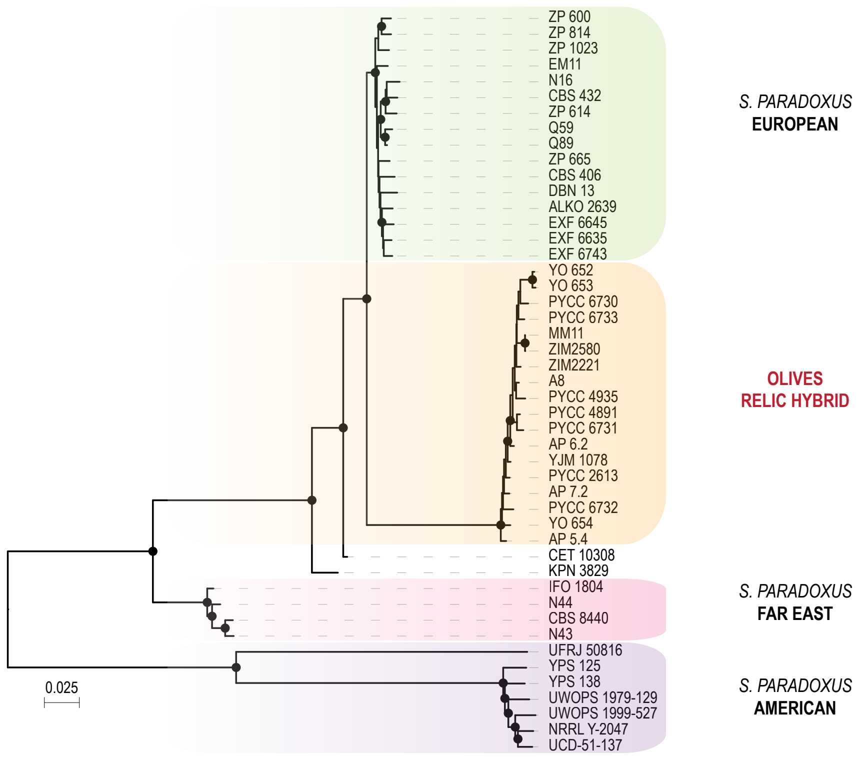

SUPPLEMENTARY FIGURE S2. Phylogenetic tree constructed with *S. paradoxus* fraction of the hybrid genomes and correspondent regions from the representatives of the European, American and Far Eastern population of *S. paradoxus*.
