# Supplementary material for: A Quasi-Domesticate Relic Hybrid Population of Saccharomyces cerevisiae × S. paradoxus Adapted to Olive Brine
Source: Front Genet. 2019 May 29;10:449. doi: 10.3389/fgene.2019.00449 (PMC6548830; doi:10.3389/fgene.2019.00449)
Supplement: Table S1 — Strains and genomes used in this study and relevant information pertaining to them. [file Table_1.pdf]

SUPPLEMENTARY TABLE S1. Strains and genomes used in this study and relevant information pertaining to them.

STRAIN	OTHER DESIGNATIONS	SUBSTRATE	ORIGIN	WGS GROUP	HETEROZIGOUS SITES (Q > 40)	SPORULATION	REGIONS			AQY1	AQY2	CNV			GENOME DATA	REFERENCE
							A	B	C			ACT1	CUP1-1	CUP1-2		
AWRI 1631	N 96	Wine	Australia	WINE	n.d.	n.d.			8/19	NF	NF	n.d	n.d	n.d	PRJNA30553	Borneman et al. 2008
AWRI 796	Active Dry Wine Yeast	Commercial wine yeast	South Africa	WINE	1041 *	n.d.		DUP		F	NF	n.d	n.d	n.d	PRJNA48559	Borneman et al. 2011
EXF 6719		Wine must	Cuber, Slovenia	WINE	2053	+			17/19	NF	NF	2	1	1	PRJEB7601	Almeida et al. 2015
ZP 641		Spontaneous red wine fermentation	Castelo de Vide, Portugal	WINE	1484	+				NF	NF	2	4	3	PRJEB7601	Almeida et al. 2015
L1374		Wine (must from País variety)	Cauquenes, Chile	WINE	1550	n.d.		5/5		NF	NF	2	2	1	SGRP2	Bergström et al. 2014
L1528		Wine (must from Cabernet variety)	Cauquenes, Chile	WINE	1334	n.d.		5/5		NF	NF	2	16	17	SGRP2	Bergström et al. 2014
Lalvin BM45	AWRI 1486	Commercial yeast (white wine)		WINE	1280	+		5/5	2/19	NF	NF	2	2	2	PRJEB19382	Almeida et al. 2017
Lalvin CY-3079	AWRI 2078	Commercial yeast (red wine)		WINE	1974	+		5/5	4/19	NF	NF	2	3	2	PRJEB19382	Almeida et al. 2017
Lalvin W15		Commercial yeast (white and red wine)		WINE	2209	+		5/5		F	NF	2	10	14	PRJEB19382	Almeida et al. 2017
PR		Pasteur Red		WINE	1715	+				NF	NF	2	2	2	PRJEB19382	Almeida et al. 2017
PYCC 4072		Commercial wine yeast (Fermivin)	Portugal	WINE	1889	+				NF	NF	2	2	3	PRJEB19382	Almeida et al. 2017
TUM V1		Bordeaux wine		WINE	1827	+				NF	NF	2	1	1	PRJEB19382	Almeida et al. 2017
Uvaferm SGV		Commercial yeast (red wine)		WINE	1734	+			4/19	NF	NF	2	1	2	PRJEB19382	Almeida et al. 2017
Uvaferm VRB		Commercial yeast (red wine)		WINE	2750	+			19/19	NF	NF	2	5	5	PRJEB19382	Almeida et al. 2017
Vin 13	AWRI 1537	Commercial wine yeast		WINE	15216 *	n.d.		DUP	2/19	F	NF	n.d	n.d	n.d	PRJNA48563	Borneman et al. 2011
VL3	B 6, AWRI 1688	Commercial wine yeast		WINE	9904 *	n.d.		5/5	8/19	NF	NF	n.d	n.d	n.d	PRJNA48565	Borneman et al. 2011
WE 372		Commercial wine yeast	Cape Town, South Africa	WINE	4354	n.d.		5/5		F	NF	2	8	10	PRJNA60199	Justin Fay, Washington University
YJM 1332		Wine	Italy	WINE	1660	n.d.			2/19	NF	NF	3	2	2	PRJNA189896	Strope et al. 2015
YJM 1336		Wine	Italy	WINE	1547	n.d.				NF	NF	n.d	n.d	n.d	PRJNA189897	Strope et al. 2015
YJM 1341	NRRL Y-12637	Grape must	South Africa	WINE	2350	n.d.				NF	NF	2	4	4	PRJNA189899	Strope et al. 2015
YJM 1415	NRRL Y-268	Wine	France	WINE	1256	n.d.		5/5	19/19	NF	NF	2	1	1	PRJNA189914	Strope et al. 2015
S8K		Bark of olive tree (variety Leccino) close to the soil	Kolomban, Slovenia	WINE	919	+		5/5	5/19	NF	NF	2	17	15	PRJEB30431	This study
YO 392		Table olive	Purchased in Tecate, Mexico	WINE	1859	+				NF	NF	2	3	3	PRJEB30431	This study
SS8		Extra virgin olive oil with macerated perforate St John's	Bozava, Dugi otok, Croatia	WINE	2565	-		5/5	5/19	NF	NF	2	19	11	PRJEB30431	This study
S21		Bark of olive tree variety, sampled close to the soil	Kolomban, Slovenia	WINE	3101	-				NF	NF	2	7	5	PRJEB30431	This study
EC 1118	Prise de Mousse	Industrial strain isolated from Champagne	France	WINE	n.d.	n.d.	15/15	5/5	19/19	NF	NF	2	2	2	PRJEA37863	Novo et al. 2009
IOC 18-2007	AWRI 2340	Commercial yeast		WINE	2181	+		5/5		NF	F	2	6	5	PRJEB19382	Almeida et al. 2017
IOC 9002		Commercial wine yeast		WINE	1224	-		5/5	4/19	NF	NF	2	11	6	PRJNA264372	Almeida et al. 2015
Lalvin QA23		Commercial wine yeast	Portugal	WINE	18861 *	+	15/15	TRIP	19/19	NF	F	2	2	2	PRJNA48561	Borneman et al. 2011
YJM 1574	AWRI 1775	Wine		WINE	1796	n.d.		5/5		NF	NF	3	7	7	PRJNA189934	Strope et al. 2015
YJM 270	CBS 2807	Wine	Slovenia	WINE	1662	n.d.			19/19	NF	F	2	1	1	PRJNA189852	Strope et al. 2015
PYCC 4074		Commercial wine yeast (Fermichamp)	Portugal	WINE	2263	+	15/15	5/5		NF	F	2	6	5	PRJEB19382	Almeida et al. 2017
PYCC 6722	CBS 5155	Wine	South Armenia	WINE	2121	+				NF	F	2	1	1	PRJEB19382	Almeida et al. 2017
PYCC 6726		Jerez-wine	Spain	WINE	4939	+				NF	F	2	1	1	PRJEB19382	Almeida et al. 2017
PYCC 6729		Jerez-wine	Armenia	WINE	2901	+				NF	F	2	1	1	PRJEB19382	Almeida et al. 2017
YO 652	Kalamata 1	Table olives (variety Kalamata)	Purchased in Seattle, Washington, USA	OLIVES	2851	+				NF	NF	2	6	6	PRJEB30431	This study
YO 653	Kalamata 2	Table olives (variety Kalamata)	Purchased in Seattle, Washington, USA	OLIVES	3342	+				NF	NF	2	4	4	PRJEB30431	This study
YO 654	Kalamata 3	Table olive brine (variety Kalamata)	Purchased in Seattle, Washington, USA	OLIVES	3466	+				NF	NF	2	8	7	PRJEB30431	This study
PYCC 4935		Table olive brine	Portalegre, Portugal	OLIVES	1713	+				NF	NF	2	1	1	PRJEB30431	This study
PYCC 4891		Table olive brine	Elvas, Portugal	OLIVES	10653	-				NF	NF	2	2	2	PRJEB30431	This study
PYCC 6730		Table olives	Spain	OLIVES	2965	+		5/5		NF	NF	2	2	1	PRJEB30431	This study
PYCC 6731		Alpechin	Spain	OLIVES	1393	+		4/5		NF	NF	2	1	1	PRJEB30431	This study
PYCC 6732	NRRL Y-6679; CBS 3081; YJM 1252	Alpechin	Spain	OLIVES	1886	-				NF	NF	2	1	1	PRJEB30431	This study
PYCC 6733		Alpechin	Spain	OLIVES	5258	-				NF	NF	2	1	1	PRJEB30431	This study
CBS 7002	PYCC 8023	Alpechin	Sevilla, Spain	OLIVES	2471	+				NF	NF	2	4	3	PRJEB30431	This study
A8		Table olive brine	Purchased in Oeiras, Portugal	OLIVES	16637	-				NF	NF	2	3	2	PRJEB30431	This study
AP 5.4		Table olive brine	Purchased in Lisbon, Portugal	OLIVES	2914	+				NF	NF	2	7	8	PRJEB30431	This study
AP 6.2		Table olive brine	Purchased in Lisbon, Portugal	OLIVES	2031	+				NF	NF	1	12	10	PRJEB30431	This study
AP 7.2		Table olive brine	Purchased in Lisbon, Portugal	OLIVES	3116	+				NF	NF	2	4	3	PRJEB30431	This study
AP 16.1		Table olive brine	Douro, Portugal	OLIVES	2618	+				NF	NF	2	20	15	PRJEB30431	This study
AP 17.1		Table olive brine	Douro, Portugal	OLIVES	2961	+				NF	NF	2	3	3	PRJEB30431	This study
MM 11		Sediment of olive oil	Kolomban, Slovenia	OLIVES	3153	-				NF	NF	2	1	1	PRJEB30431	This study
ZIM 2221		Olive oil (variety Oblica)	Split, Croatia	OLIVES	14951	-		5/5		NF	NF	2	8	10	PRJEB30431	This study
ZIM 2580		Sediment of olive oil	Kolomban, Slovenia	OLIVES	3117	+				NF	NF	2	1	1	PRJEB30431	This study
S27		Ripe olive (variety Belica)	Kolomban, Slovenia	OLIVES	3217	-				NF	NF	2	3	2	PRJEB30431	This study
PYCC 2708		Swine rectum	Portugal	OLIVES	3559	+				NF	NF	2	7	8	PRJEB30431	This study
PYCC 2613	CBS 2909; NRRL YB-6041	Human feces	Portugal	OLIVES	6783	+				NF	NF	2	2	4	PRJEB30431	This study
NRRL-Y 12658	CBS 4411; PYCC 8033	Pig rectal contents	unknown	OLIVES	2850	+				NF	NF	2	17	16	PRJEB30431	This study
YJM 248	NRRL Y-12659; CBS 2910; PYCC 8034	Human feces	Portugal	OLIVES	6185	n.d.				NF	NF	2	5	5	SRR800854	Strope et al. 2015
YJM 1078	NRRL YB-4348; PYCC 2625; PYCC 8028	Human feces	Portugal	OLIVES	2752	n.d.				NF	NF	3	6	5	SRR800768	Strope et al. 2015
PYR 4b	DBQ 26	*Quercus pubescens*	Halkidiki, Greece	MO	2738	n.d.				F	F	2	1	1	PRJEB7601	Almeida et al. 2015
EXF 7200		*Quercus robur*	Jasenovo Polje, Montenegro	MO	3001	n.d.				F	F	2	1	1	PRJNA264372	Almeida et al. 2015

HUN 9.1s1	DBS 14	Oak	Hungary	MO	2745	n.d.				F	F	2	1	1	PRJEB7601	Almeida et al. 2015
ZP 541		*Fagus sylvatica*	Adagoi, Portugal	MO	2684	+				F	F	2	1	1	PRJEB7601	Almeida et al. 2015
ZP 570		*Fraxinus* sp.	Paul Boquilobo, Portugal	MO	2813	+				F	F	2	1	1	PRJEB7601	Almeida et al. 2015
ZP 848		*Quercus ilex*	Alter do Chão, Portugal	MO	2656	n.d.				F	F	3	1	1	PRJEB7675	Almeida et al. 2015
PYCC 4226		Commercial baker's yeast		BREAD	30081	-		4/5	2/19	NF	NF	2	1	2	ERS1108635	Gonçalves et al. 2016
Platinum		Commercial baker's yeast		BREAD	42278	-		4/5	15/19	NF	NF	2	1	1	ERS1108633	Gonçalves et al. 2016
AP 13.1		Baker's yeast	Portugal	BREAD	53091	-		5/5	17/19	NF	NF	2	2	2	PRJEB24932	Barbosa et al. 2018
TUM 381		Belgian beer	Belgium	BEER 1 - WHEAT	30358	-				NF	F				ERS1108612	Gonçalves et al. 2016
TUM 508		Irish ale / stout	Ireland	BEER 1 - BRITISH	44826	-				NF	NF				ERS1108630	Gonçalves et al. 2016
TUM 208		Alt beer	Rhineland-Palatinate, Germany	BEER 1 - GERMAN	33689	-				NF	NF				ERS1108617	Gonçalves et al. 2016
CBS 1598		Sake moto	Nakazawa, Japan	SAKE	3517	-				NF	NF	2	3	1	ERS1108639	Gonçalves et al. 2016
UC5	UCD612	Sene sake	Kurashi, Japan	SAKE	n.d.	n.d.				NF	NF				PRJNA60197	Justin Fay, Washington University
Kyokai7		Sake yeast	Japan	SAKE	n.d.	n.d.				NF	NF				PRJNA45827	Akao et al. 2011
A38		Olive collected from olive tree	Alqueva, Portugal	SAKE	2747	-				NF	NF	1	4	4	PRJEB30431	This study
TUM 127	progeny of TUM 68	Wheat beer	Freising-Weihenstephan, Germany	SAKE	5979	-				NF	NF				ERS1108644	Gonçalves et al. 2016
NRRL-YB-4084		Coconut sap	Philippines	PHILIPPINES	10363	-				NF	F				ERS1108646	Gonçalves et al. 2016
YJM 1400	NRRL YB-4081	Guava (fruit)	Philippines	PHILIPPINES	2960	n.d.				NF	NF				PRJNA189911	Strope et al. 2015
ZP 779		*Quercus acutissima*	Okayama prefecture, Japan	NA & JP	3509	+				F	F				PRJEB7601	Almeida et al. 2015
ZP 781		*Quercus serrata*	Okayama prefecture, Japan	NA & JP	3333	+				F	F				PRJEB7601	Almeida et al. 2015
YPS 128		*Quercus alba*	Pennsylvania, USA	NA & JP	3118	n.d.				F	F				SGRP2	Bergström et al. 2014
YPS 163		*Quercus rubra*	Pennsylvania, USA	NA & JP	2673	n.d.				F	F				PRJNA28813	Doniger et al. 2008
ZP 651		*Quercus acutissima*	Chiba prefecture, Japan	NA & JP	3623	+				F	F				PRJEB7601	Almeida et al. 2015
ZP 656		*Quercus acuta*	Chiba prefecture, Japan	NA & JP	3723	+				F	F				PRJEB7601	Almeida et al. 2015
PW5		Raphia palm wine	Aba, Abia state, Nigeria	WEST AFRICA	1175	n.d.				NF	F				PRJNA60181	Justin Fay, Washington University
YJM 1248	NRRL Y-1546	Bili wine from *Osbeckia grandiflora*	West Africa	WEST AFRICA	2600	n.d.				NF	F				PRJNA189888	Strope et al. 2015
YJM 1439	NCYC 110	Ginger beer from *Z. officinale*	West Africa	WEST AFRICA	2129	n.d.				NF	F				PRJNA189920	Strope et al. 2015
UWOPS 03-461-4		Nectar of Bertram palm	Malaysia	MALAYSIA	18636	n.d.				NF	NF				SGRP2	Bergström et al. 2014
UWOPS 03-433-3		Nectar of Bertram palm	Malaysia	MALAYSIA	1908	+				--	--				ERS1108647	Gonçalves et al. 2016
YJM 1447	UWOPS 05-227.2	Bertram palm	Malaysia	MALAYSIA	n.d.	n.d.				NF	NF	n.d.	n.d	n.d.	PRJNA189923	Strope et al. 2015
A44		Olive collected from olive tree	Alqueva, Portugal	MOSAIC	3768	+				NF	NF	2	9	6	PRJEB30431	This study
A5		Olive collected from olive tree (variety Gaeta)	Mação, Portugal	MOSAIC	4551	+				F	NF	2	2	1	PRJEB30431	This study

* values taken from the literature (see reference)

MO, Mediterranean oaks
NA & JP, North America and Japan

F Functional
NF Non Functional

References:

Akao et al. 2011. Whole-genome sequencing of sake yeast Saccharomyces cerevisiae Kyokai no. 7. DNA Res. 18, 423-434.

Almeida et al. 2015. A population genomic insigth into the Mediterranean origins of wine yeast domestication. Mol. Eco. 24, 5412-5427.

Almeida et al. 2017. Adaptive divergence in wine yeasts and their wild relatives suggests a prominent role for introgressions and rapid evolution at noncoding sites. Mol. Ecol. 26, 2167-2182.

Barbosa et al. 2018. Multiple rounds of artificial selection promote microbe secondary domestication – the case of cachaça yeasts. GBE 10,1939-1955.

Bergström et al. 2014. A high-definition view of functional genetic variation from natural yeast genomes. Mol Biol Evol 31, 872–888.

Borneman et al. 2008. Comparative genome analysis of a Saccharomyces cerevisiae wine strain. FEMS Yeast Res. 8, 1185-1195.

Borneman et al. 2011. Whole-genome comparison reveals novel genetic elements that characterize the genome of industrial strains of Saccharomyces cerevisiae. PLoS Genet. 7, E1001287.

Doniger et al. 2008. A catalog of neutral and deleterious polymorphism in yeast. PLoS Genet 4, e1000183.

Gonçalves et al. 2016. Distinct domestication trajectories in top-fermenting beer yeasts and wine yeasts. Curr Biol 26, 2750-2761.

Novo et al. 2009. Eukaryote-to-eukaryote gene transfer events revealed by the genome sequence of the wine yeast Saccharomyces cerevisiae EC1118. PNAS 106, 16333-16338.

Strope et al. 2015. The 100-genomes strains, an S. cerevisiae resource that illuminates its natural phenotypic and genotypic variation and emergence as an opportunistic pathogen. Genome Res. 25, 762-774.
