# Supplementary material for: A Quasi-Domesticate Relic Hybrid Population of Saccharomyces cerevisiae × S. paradoxus Adapted to Olive Brine
Source: Front Genet. 2019 May 29;10:449. doi: 10.3389/fgene.2019.00449 (PMC6548830; doi:10.3389/fgene.2019.00449)
Supplement: Table S2 — Gene ontology (GO) analysis of the S. paradoxus sub-genome of relict hybrid strains. [file Table_2.pdf]

SUPPLEMENTARY TABLE S2. Gene Ontology (GO) analysis of the *S. paradoxus* sub-genome of relic hybrid strains.

OLIVE BRINE | ALPECHIN / OLIVE OIL | OLIVE TREE | INTESTINAL TRACT

GO TERM	GENE ONTOLOGY TERM (P-VALUE < 0.01)	PYCC 4935	PYCC 4891	A8	AP 5.4	AP 6.2	AP 7.2	AP 16.1	AP 17.1	YO 652	YO 653	YO 654	PYCC 6730	PYCC 6731	PYCC 6732	PYCC 6733	MM 11	ZIM 2221	ZIM 2580	S27	PYCC 2613	PYCC 2708	YJM 248	YJM 1078	SHARED
PROCESS	Fungal-type cell wall organization or biogenesis																								
	Fungal-type cell wall organization																								
	External encapsulating structure organization																								
	Cell wall organization																								
	Cell wall organization or biogenesis																								
	Carbohydrate transmembrane transport																								
	Carbohydrate transport																								
	Glucose transport																								
	Fructose transport																								
	Mannose transport																								
	Monosaccharide transport																								
	Monosaccharide transmembrane transport																								
	Glucose transmembrane transport																								
	Hexose transmembrane transport																								
	Hexose transport																								
FUNCTION	Glucose transmembrane transporter activity																								
	Monosaccharide transmembrane transporter activity																								
	Hexose transmembrane transporter activity																								
	Carbohydrate transmembrane transporter activity																								
	Mannose transmembrane transporter activity																								
	Fructose transmembrane transporter activity																								
	Glucose transmembrane transporter activity																								
	Sugar transmembrane transporter activity																								
	Symporter activity																								
	Solute proton symporter activity																								
	Solute cation symporter activity																								
	Sugar proton symporter activity																								
	Cation sugar symporter activity																								
	Structural constituent of cell wall																								
	Transporter activity																								
	Transmembrane transporter activity																								
COMPONENT	External encapsulating structure																								
	Cell wall																								
	Fungal-type cell wall																								
	Cell periphery																								
	Plasma membrane																								
	Plasma membrane part																								
	Membrane part																								
	Cell part																								
	Cell																								
	Integral component of plasma membrane																								
	Intrinsic component of plasma membrane																								
	Intrinsic component of membrane																								
	Anchored component of membrane																								
	Extracellular region																								
	Membrane																								
	**Number of Genes Analysed**	251	254	314	288	246	237	249	275	193	216	282	227	241	271	259	269	269	270	236	268	250	259	244	103
