# Supplementary material for: A Quasi-Domesticate Relic Hybrid Population of Saccharomyces cerevisiae × S. paradoxus Adapted to Olive Brine
Source: Front Genet. 2019 May 29;10:449. doi: 10.3389/fgene.2019.00449 (PMC6548830; doi:10.3389/fgene.2019.00449)
Supplement: Table S3 — Cfu/ml counts per strain and per replicate for the growth and survival in olive brine experiment shown in Figure 2. [file Table_3.pdf]

SUPPLEMENTARY TABLE S3. Cfu/ml counts per strain and per replicate for the growth and survival in olive brine experiment shown in Figure 2.

Strain	Replicate	Days 0	8	15	24	34	41	49	57	70
YO 654	1	9.8×10^4	3.1×10^6	4.1×10^6	3.2×10^6	3.3×10^6	3.0×10^6	3.5×10^6	1.9×10^6	3.2×10^6
	2	9.6×10^4	3.1×10^6	3.9×10^6	3.9×10^6	3.4×10^6	2.5×10^6	3.2×10^6	2.8×10^6	3.7×10^6
ZIM 2580	1	9.8×10^4	4.2×10^6	2.1×10^6	1.3×10^6	8.1×10^5	6.7×10^5	5.6×10^5	9.6×10^5	7.5×10^5
	2	7.5×10^4	1.8×10^6	1.96×10^6	1.5×10^6	7.3×10^5	1.3×10^6	8.2×10^5	8.0×10^5	7.1×10^5
AP 7.2	1	1.1×10^5	8.1×10^6	5.0×10^6	3.9×10^6	2.8×10^6	6.4×10^6	4.2×10^6	5.0×10^6	3.9×10^6
	2	1.1×10^5	3.5×10^6	3.4×10^6	5.3×10^6	5.2×10^6	3.8×10^6	4.2×10^6	4.9×10^6	3.7×10^6
PYCC 4891	1	1.2×10^5	2.0×10^6	2.0×10^6	1.8×10^6	3.9×10^6	2.2×10^6	2.6×10^6	3.7×10^6	3.1×10^6
	2	1.4×10^5	2.5×10^6	3.0×10^6	1.5×10^6	3.0×10^6	3.3×10^6	1.4×10^6	2.5×10^6	2.6×10^6
AP 5.4	1	1.2×10^5	3.4×10^6	3.5×10^6	3.5×10^6	3.4×10^6	2.1×10^6	1.8×10^6	1.7×10^6	1.5×10^6
	2	1.2×10^5	4.4×10^6	4.5×10^6	4.1×10^6	4.4×10^6	2.1×10^6	1.9×10^6	2.8×10^6	2.0×10^6
PYCC 6732	1	1.0×10^5	2.5×10^6	2.3×10^6	3.6×10^6	4.9×10^6	2.9×10^6	1.6×10^6	2.1×10^6	2.3×10^6
	2	1.2×10^5	2.2×10^6	2.4×10^6	2.4×10^6	2.3×10^6	3.6×10^6	1.9×10^6	2.6×10^6	2.9×10^6
Lalvin W15	1	1.2×10^5	1.0×10^6	9.6×10^5	4.1×10^5	9.1×10^4	9.1×10^4	6.4×10^4	7.6×10^4	5.5×10^4
	2	1.9×10^5	9.1×10^5	1.3×10^6	4.5×10^5	1.8×10^5	1.6×10^5	2.3×10^5	3.4×10^5	1.3×10^5
AWRI 1631	1	9.7×10^4	3.87×10^5	2.2×10^5	2.9×10^4	1.2×10^4	1.6×10^4	2.2×10^4	1.8×10^4	1.3×10^4
	2	1.9×10^5	6.5×10^5	2.0×10^5	2.8×10^4	1.8×10^4	2.2×10^4	1.3×10^4	1.6×10^4	9.6×10^3
PR	1	1.0×10^5	4.8×10^5	2.4×10^5	1.2×10^5	2.6×10^5	2.4×10^5	1.4×10^5	1.5×10^5	1.8×10^5
	2	1.4×10^5	1.5×10^5	2.2×10^5	1.9×10^5	1.8×10^5	n.d.	n.d.	n.d.	n.d.
Uvaferm VRB	1	9.6×10^4	2.5×10^5	1.5×10^5	6.0×10^4	2.6×10^4	3.9×10^4	2.9×10^4	6.7×10^4	3.7×10^4
	2	1.0×10^5	2.3×10^5	2.2×10^5	2.5×10^4	2.9×10^4	2.5×10^4	1.2×10^4	3.3×10^4	1.3×10^4
PYCC 4072	1	1.2×10^5	1.5×10^5	9.3×10^4	3.5×10^4	2.9×10^4	6.4×10^4	2.4×10^4	4.4×10^4	9.4×10^4
	2	1.8×10^5	2.6×10^5	1.0×10^5	5.5×10^4	5.5×10^4	7.1×10^4	4.7×10^4	9.0×10^4	3.9×10^4
TUM V1	1	2.1×10^5	2.8×10^5	4.6×10^4	5.2×10^4	2.6×10^4	1.7×10^4	2.3×10^4	2.1×10^4	4.5×10^4
	2	1.4×10^5	2.0×10^5	9.7×10^4	1.0×10^4	1.6×10^4	1.4×10^4	6.9×10^4	5.4×10^4	1.5×10^4
