# Supplementary material for: A Quasi-Domesticate Relic Hybrid Population of Saccharomyces cerevisiae × S. paradoxus Adapted to Olive Brine
Source: Front Genet. 2019 May 29;10:449. doi: 10.3389/fgene.2019.00449 (PMC6548830; doi:10.3389/fgene.2019.00449)
Supplement: DATASET S1 — Gene content of the S. paradoxus sub-genome of relict hybrid strains. [file Data_Sheet_1.pdf]

SUPPLEMENTARY DATASET S1. Gene content of the *S. paradoxus* sub-genome of relic hybrid strains.

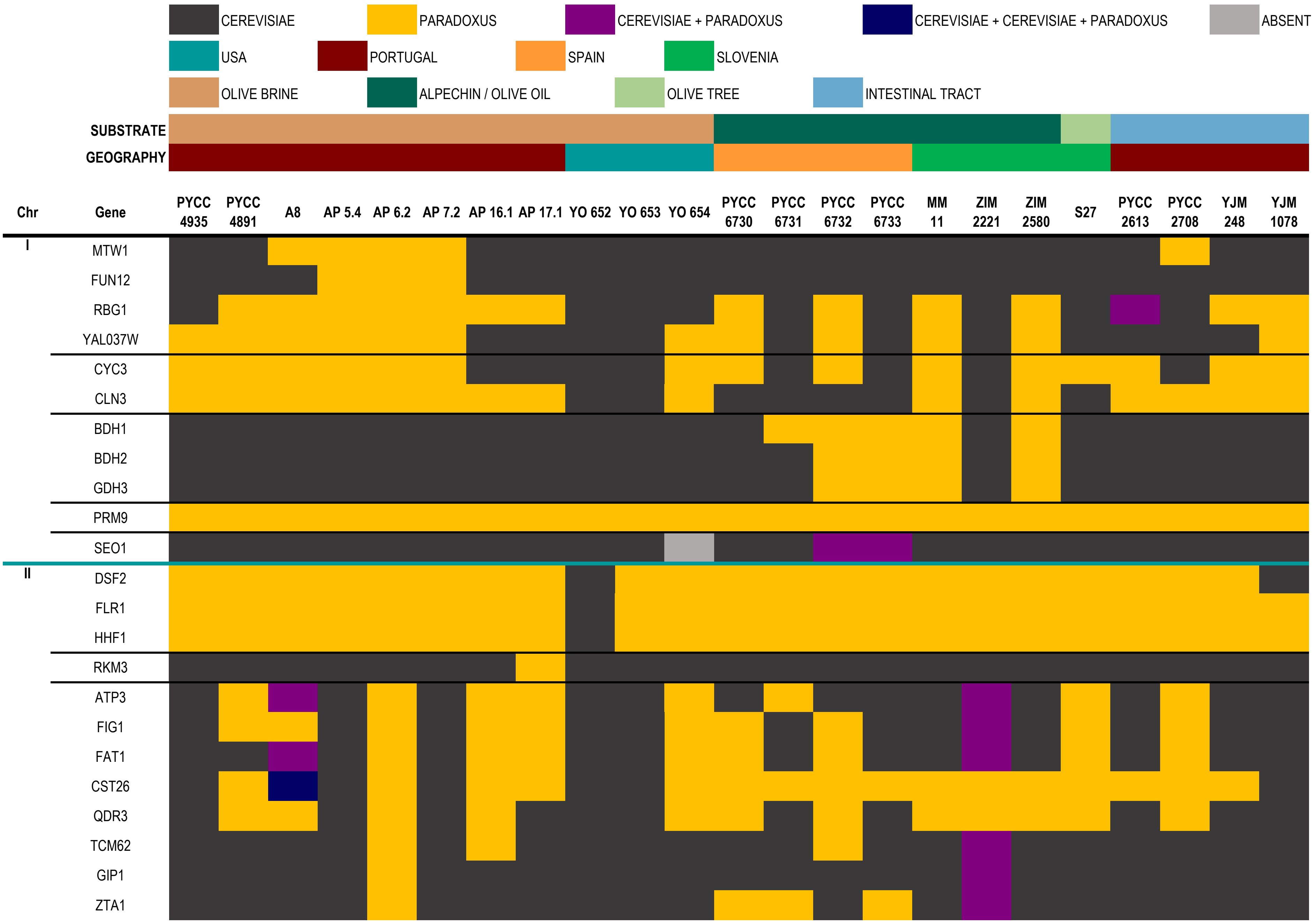

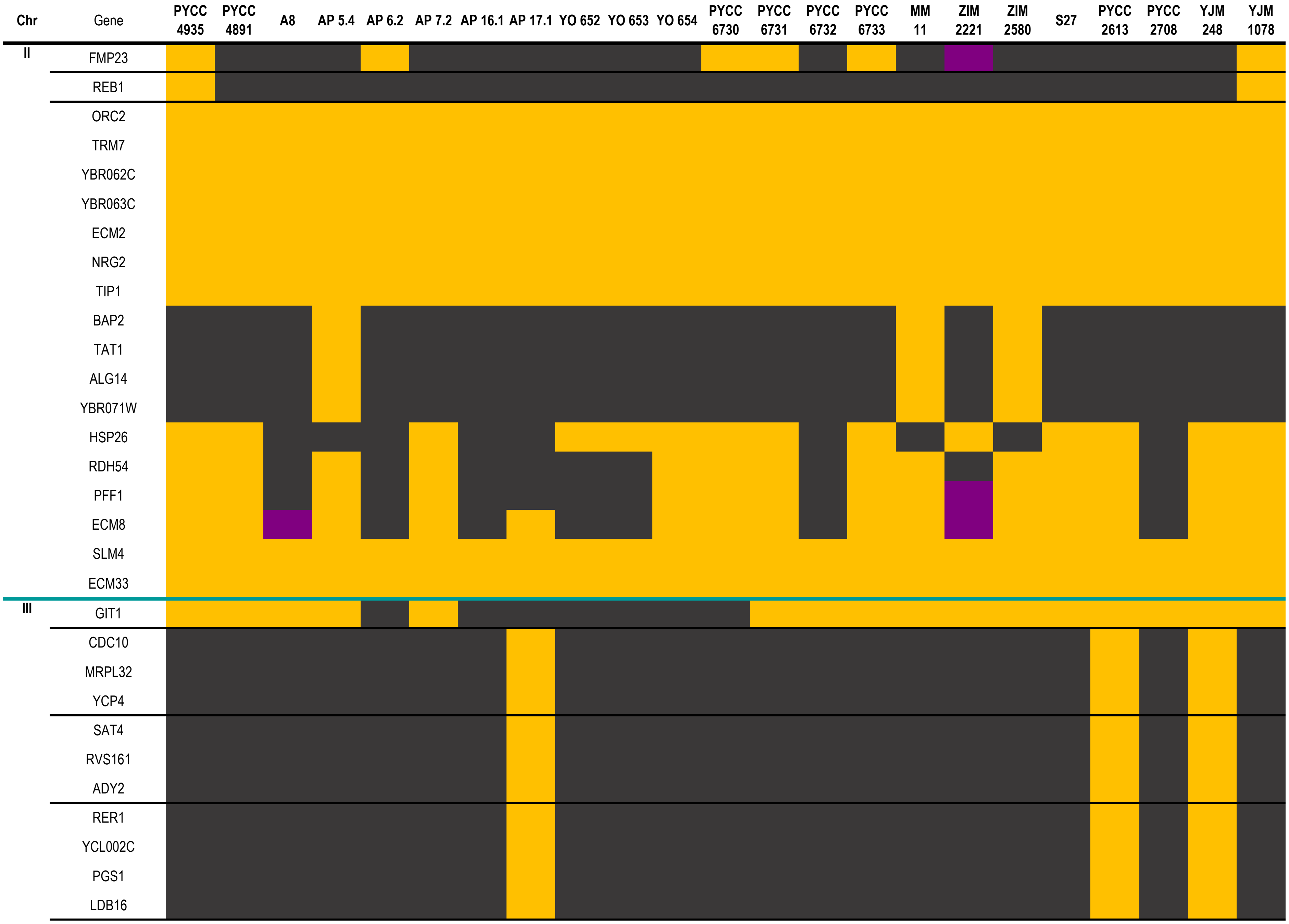
Chr
Gene
PYCC 4935
PYCC 4891
A8
AP 5.4
AP 6.2
AP 7.2
AP 16.1
AP 17.1
YO 652
YO 653
YO 654
PYCC 6730
PYCC 6731
PYCC 6732
PYCC 6733
MM 11
ZIM 2221
ZIM 2580
S27
PYCC 2613
PYCC 2708
YJM 248
YJM 1078
II
FMP23
REB1
ORC2
TRM7
YBR062C
YBR063C
ECM2
NRG2
TIP1
BAP2
TAT1
ALG14
YBR071W
HSP26
RDH54
PFF1
ECM8
SLM4
ECM33
III
GIT1
CDC10
MRPL32
YCP4
SAT4
RVS161
ADY2
RER1
YCL002C
PGS1
LDB16

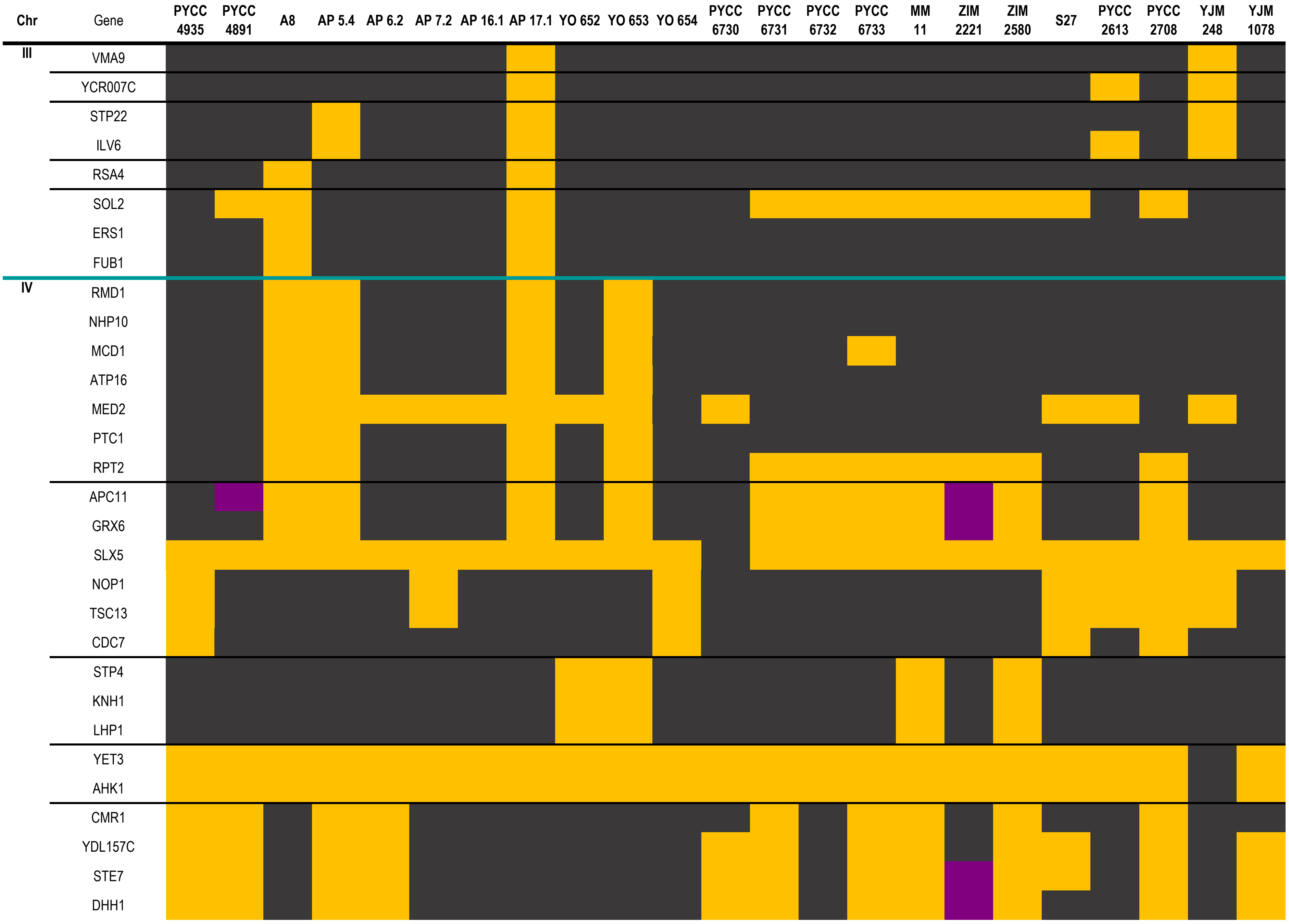
Chr
Gene
PYCC 4935
PYCC 4891
A8
AP 5.4
AP 6.2
AP 7.2
AP 16.1
AP 17.1
YO 652
YO 653
YO 654
PYCC 6730
PYCC 6731
PYCC 6732
PYCC 6733
MM 11
ZIM 2221
ZIM 2580
S27
PYCC 2613
PYCC 2708
YJM 248
YJM 1078
III
VMA9
YCR007C
STP22
ILV6
RSA4
SOL2
ERS1
FUB1
IV
RMD1
NHP10
MCD1
ATP16
MED2
PTC1
RPT2
APC11
GRX6
SLX5
NOP1
TSC13
CDC7
STP4
KNH1
LHP1
YET3
AHK1
CMR1
YDL157C
STE7
DHH1

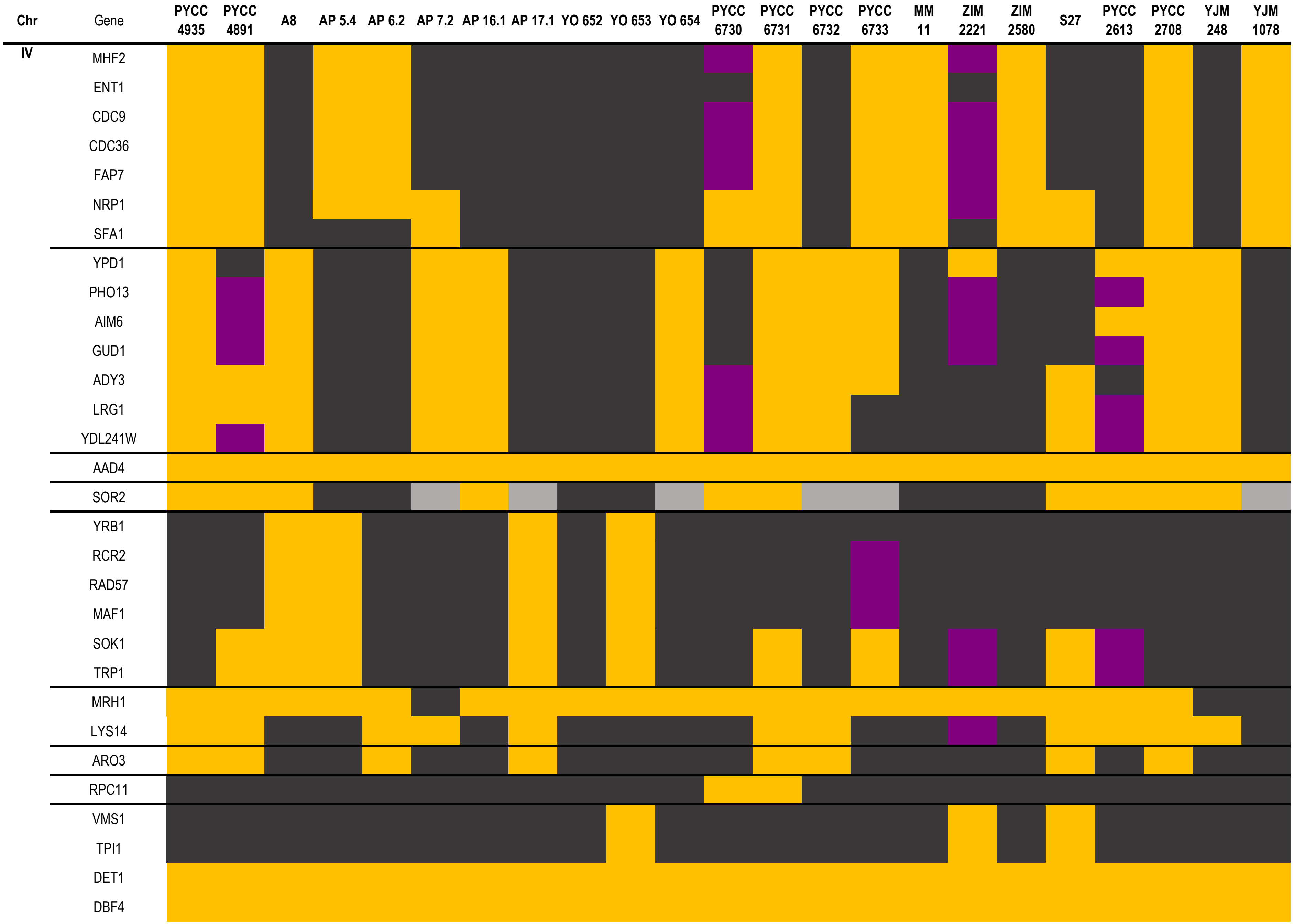
Chr
Gene
PYCC 4935
PYCC 4891
A8
AP 5.4
AP 6.2
AP 7.2
AP 16.1
AP 17.1
YO 652
YO 653
YO 654
PYCC 6730
PYCC 6731
PYCC 6732
PYCC 6733
MM 11
ZIM 2221
ZIM 2580
S27
PYCC 2613
PYCC 2708
YJM 248
YJM 1078
IV
MHF2
ENT1
CDC9
CDC36
FAP7
NRP1
SFA1
YPD1
PHO13
AIM6
GUD1
ADY3
LRG1
YDL241W
AAD4
SOR2
YRB1
RCR2
RAD57
MAF1
SOK1
TRP1
MRH1
LYS14
ARO3
RPC11
VMS1
TPI1
DET1
DBF4

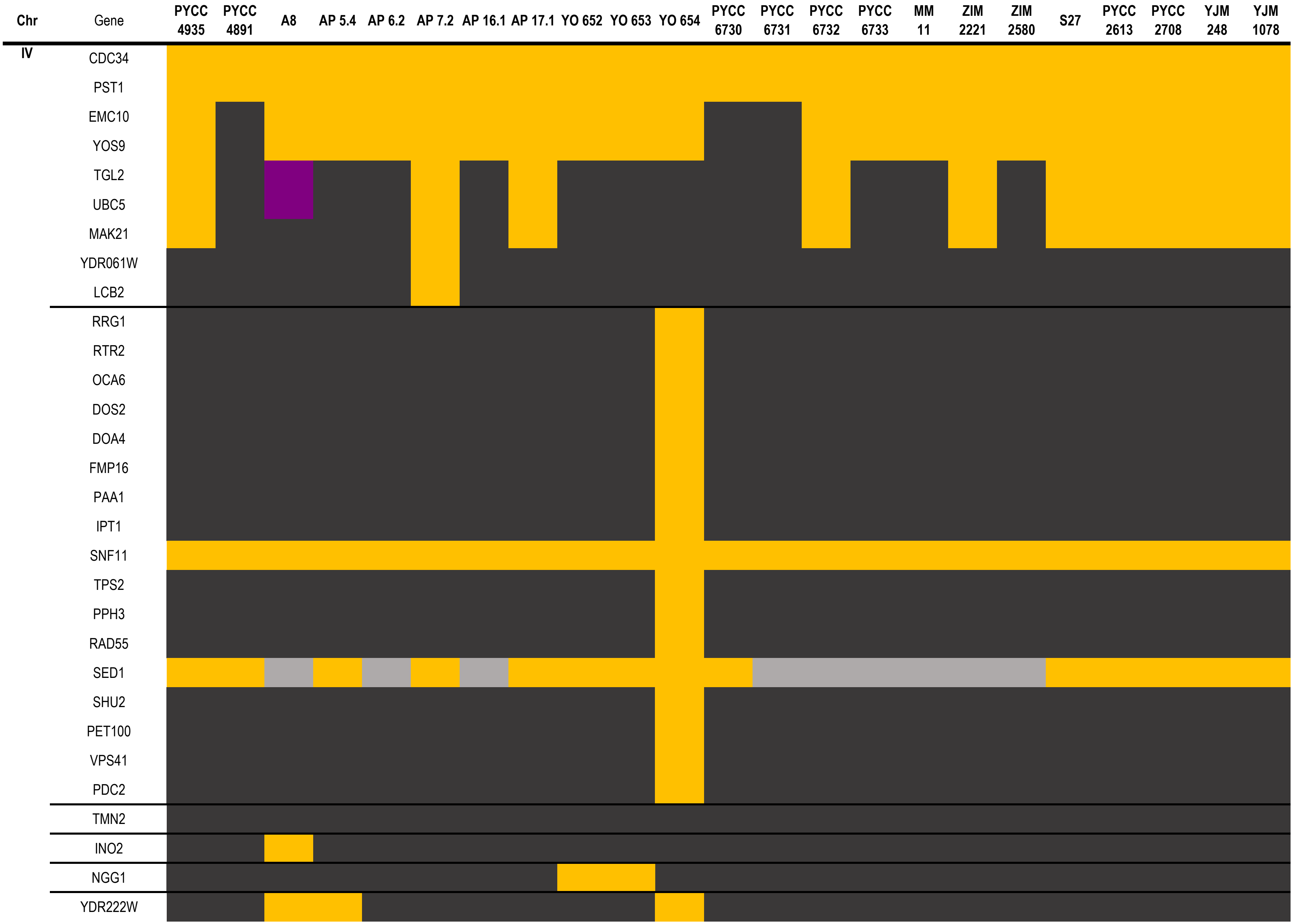
Chr
Gene
PYCC 4935
PYCC 4891
A8
AP 5.4
AP 6.2
AP 7.2
AP 16.1
AP 17.1
YO 652
YO 653
YO 654
PYCC 6730
PYCC 6731
PYCC 6732
PYCC 6733
MM 11
ZIM 2221
ZIM 2580
S27
PYCC 2613
PYCC 2708
YJM 248
YJM 1078
IV
CDC34
PST1
EMC10
YOS9
TGL2
UBC5
MAK21
YDR061W
LCB2
RRG1
RTR2
OCA6
DOS2
DOA4
FMP16
PAA1
IPT1
SNF11
TPS2
PPH3
RAD55
SED1
SHU2
PET100
VPS41
PDC2
TMN2
INO2
NGG1
YDR222W

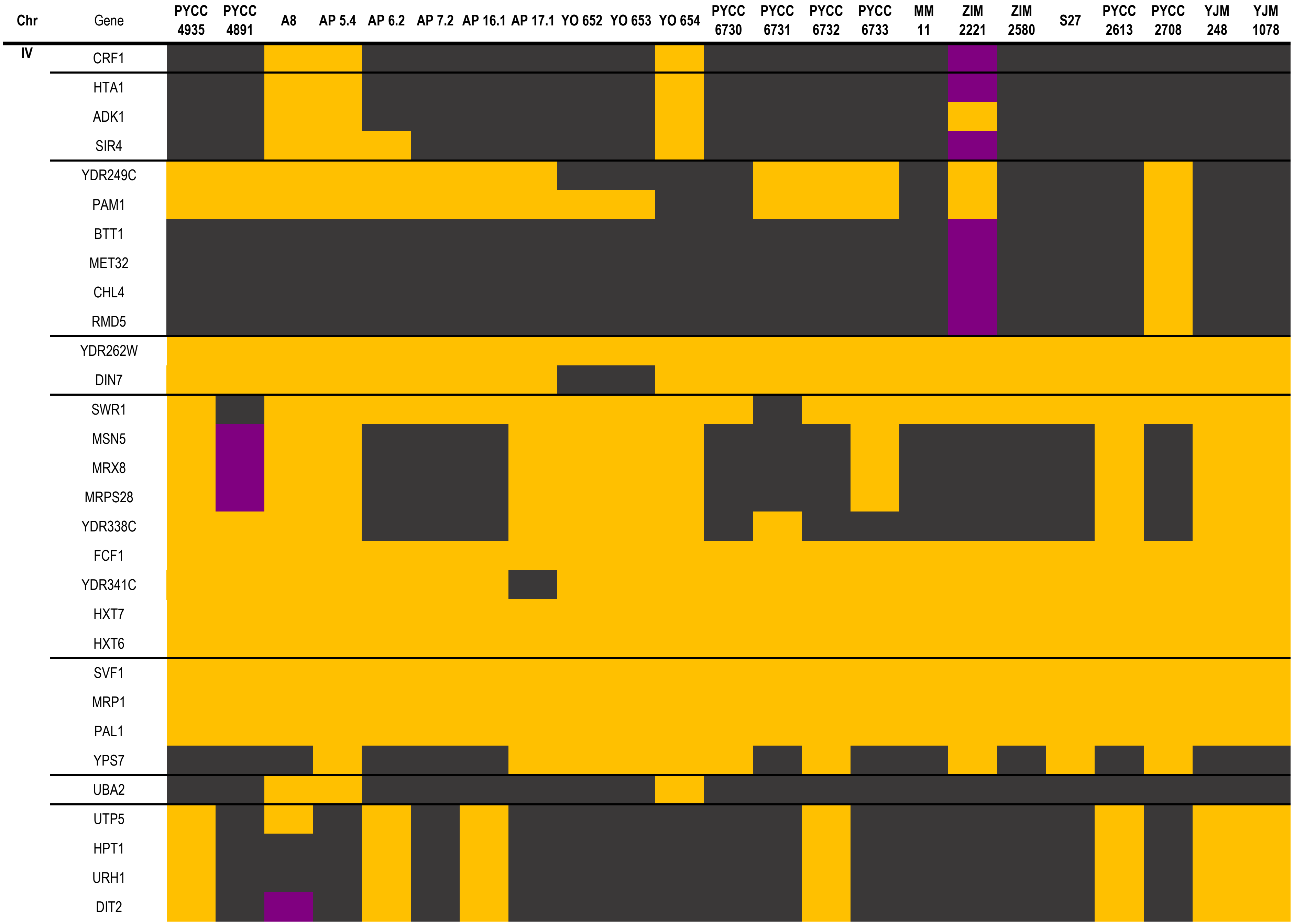
Chr
Gene
PYCC 4935
PYCC 4891
A8
AP 5.4
AP 6.2
AP 7.2
AP 16.1
AP 17.1
YO 652
YO 653
YO 654
PYCC 6730
PYCC 6731
PYCC 6732
PYCC 6733
MM 11
ZIM 2221
ZIM 2580
S27
PYCC 2613
PYCC 2708
YJM 248
YJM 1078
IV
CRF1
HTA1
ADK1
SIR4
YDR249C
PAM1
BTT1
MET32
CHL4
RMD5
YDR262W
DIN7
SWR1
MSN5
MRX8
MRPS28
YDR338C
FCF1
YDR341C
HXT7
HXT6
SVF1
MRP1
PAL1
YPS7
UBA2
UTP5
HPT1
URH1
DIT2

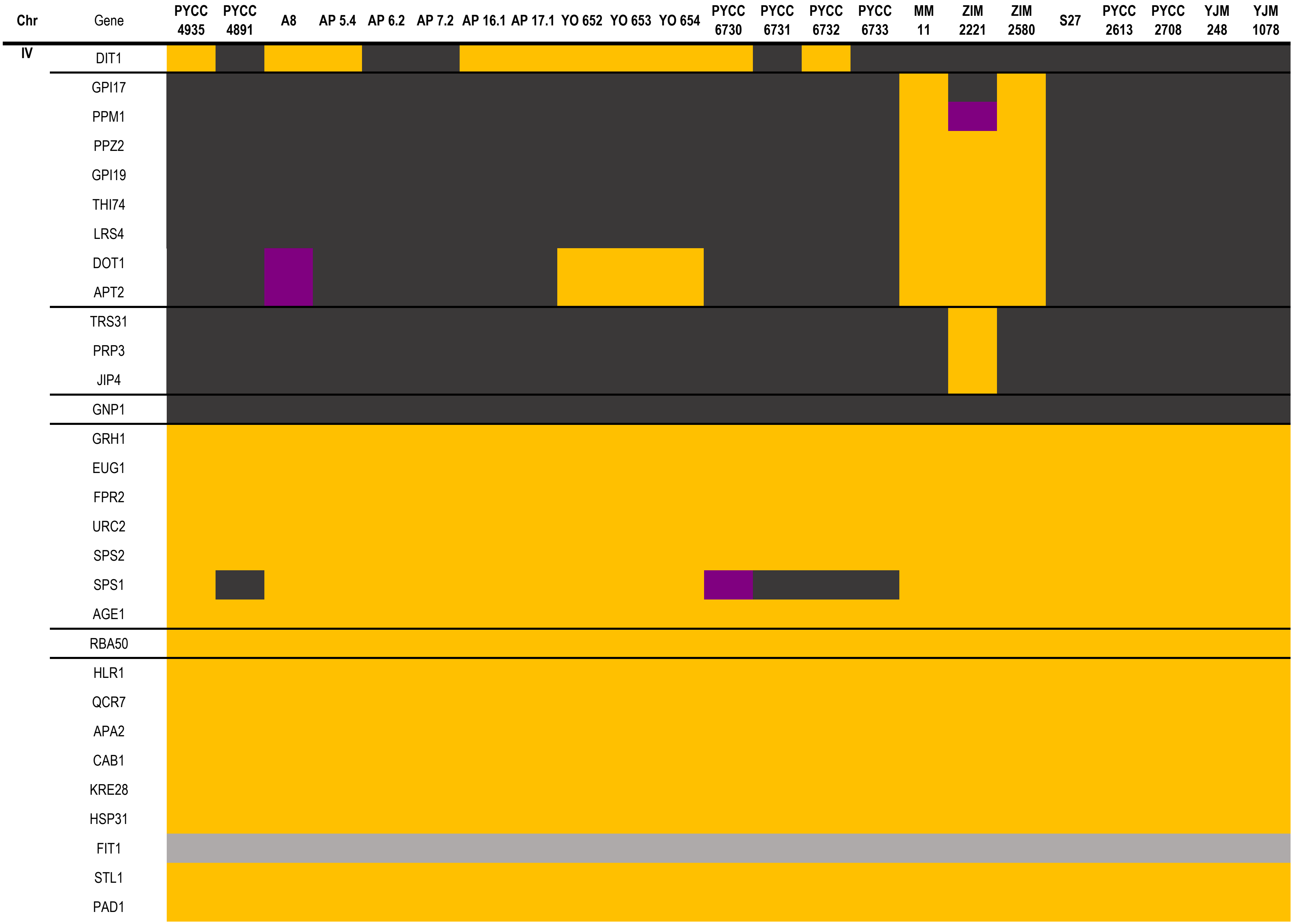
Chr
Gene
PYCC 4935
PYCC 4891
A8
AP 5.4
AP 6.2
AP 7.2
AP 16.1
AP 17.1
YO 652
YO 653
YO 654
PYCC 6730
PYCC 6731
PYCC 6732
PYCC 6733
MM 11
ZIM 2221
ZIM 2580
S27
PYCC 2613
PYCC 2708
YJM 248
YJM 1078
IV
DIT1
GPI17
PPM1
PPZ2
GPI19
THI74
LRS4
DOT1
APT2
TRS31
PRP3
JIP4
GNP1
GRH1
EUG1
FPR2
URC2
SPS2
SPS1
AGE1
RBA50
HLR1
QCR7
APA2
CAB1
KRE28
HSP31
FIT1
STL1
PAD1

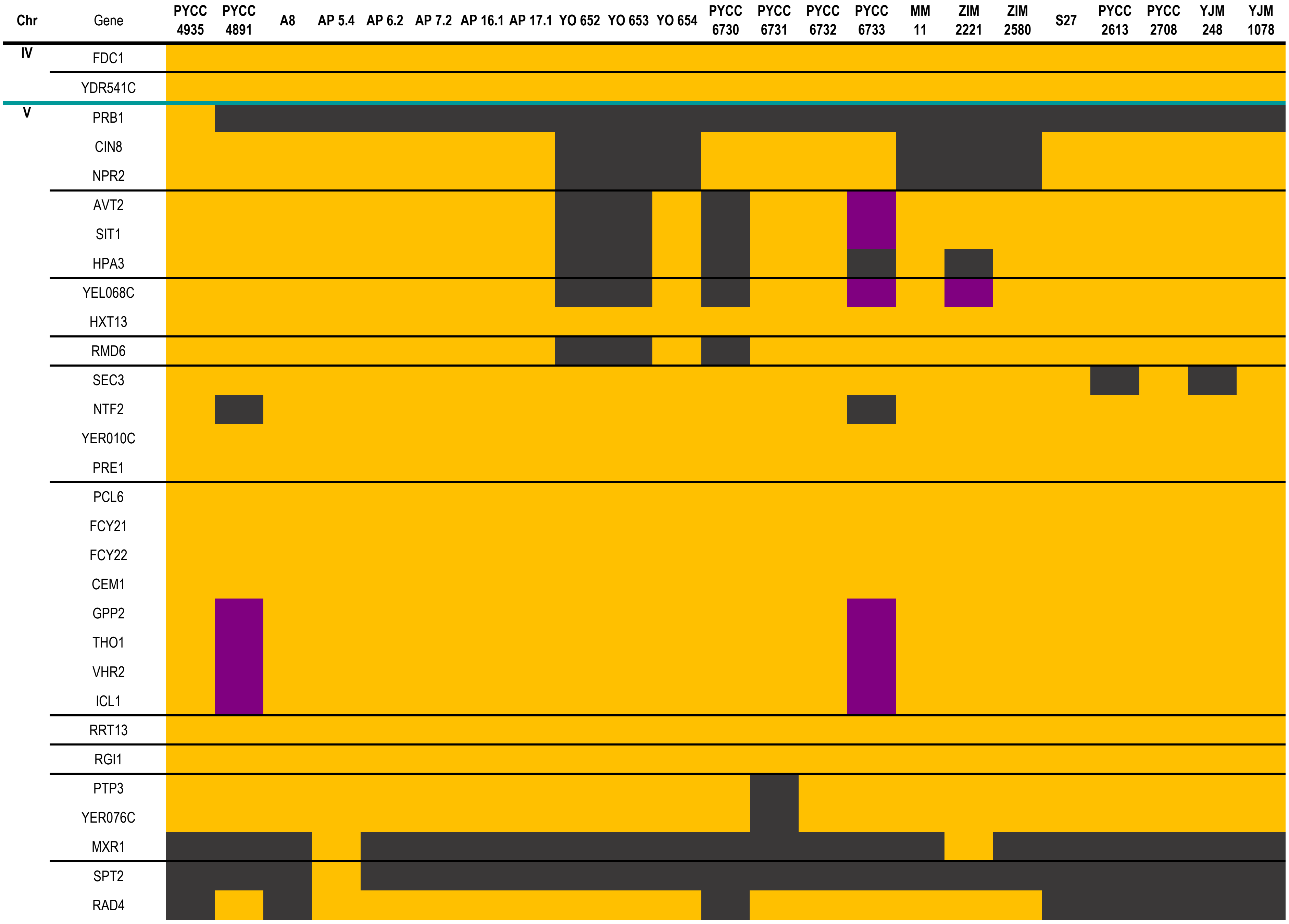
Chr
Gene
PYCC 4935
PYCC 4891
A8
AP 5.4
AP 6.2
AP 7.2
AP 16.1
AP 17.1
YO 652
YO 653
YO 654
PYCC 6730
PYCC 6731
PYCC 6732
PYCC 6733
MM 11
ZIM 2221
ZIM 2580
S27
PYCC 2613
PYCC 2708
YJM 248
YJM 1078
IV
FDC1
YDR541C
V
PRB1
CIN8
NPR2
AVT2
SIT1
HPA3
YEL068C
HXT13
RMD6
SEC3
NTF2
YER010C
PRE1
PCL6
FCY21
FCY22
CEM1
GPP2
THO1
VHR2
ICL1
RRT13
RGI1
PTP3
YER076C
MXR1
SPT2
RAD4

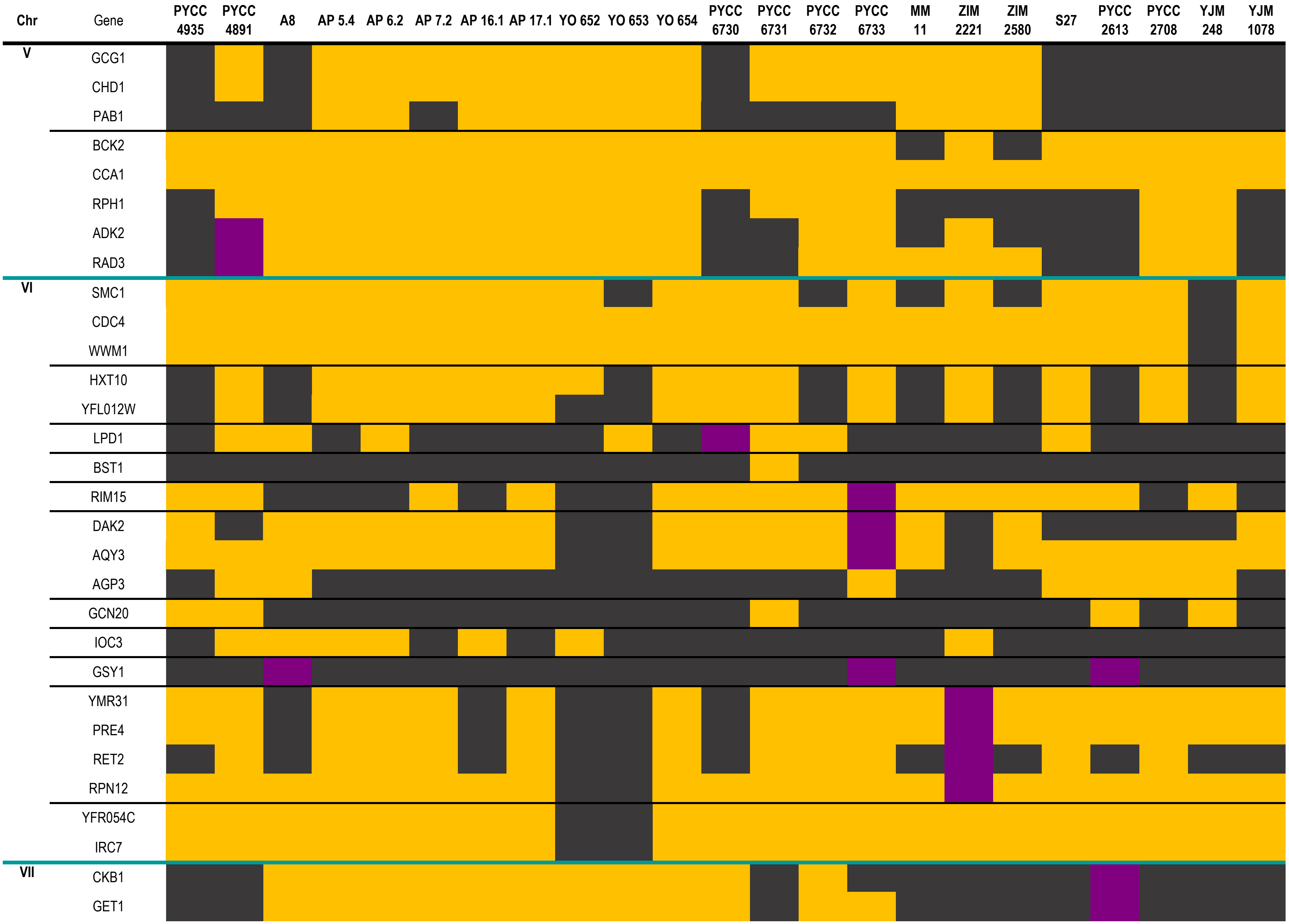
Chr
Gene
PYCC 4935
PYCC 4891
A8
AP 5.4
AP 6.2
AP 7.2
AP 16.1
AP 17.1
YO 652
YO 653
YO 654
PYCC 6730
PYCC 6731
PYCC 6732
PYCC 6733
MM 11
ZIM 2221
ZIM 2580
S27
PYCC 2613
PYCC 2708
YJM 248
YJM 1078
V
GCG1
CHD1
PAB1
BCK2
CCA1
RPH1
ADK2
RAD3
VI
SMC1
CDC4
WWM1
HXT10
YFL012W
LPD1
BST1
RIM15
DAK2
AQY3
AGP3
GCN20
IOC3
GSY1
YMR31
PRE4
RET2
RPN12
YFR054C
IRC7
VII
CKB1
GET1

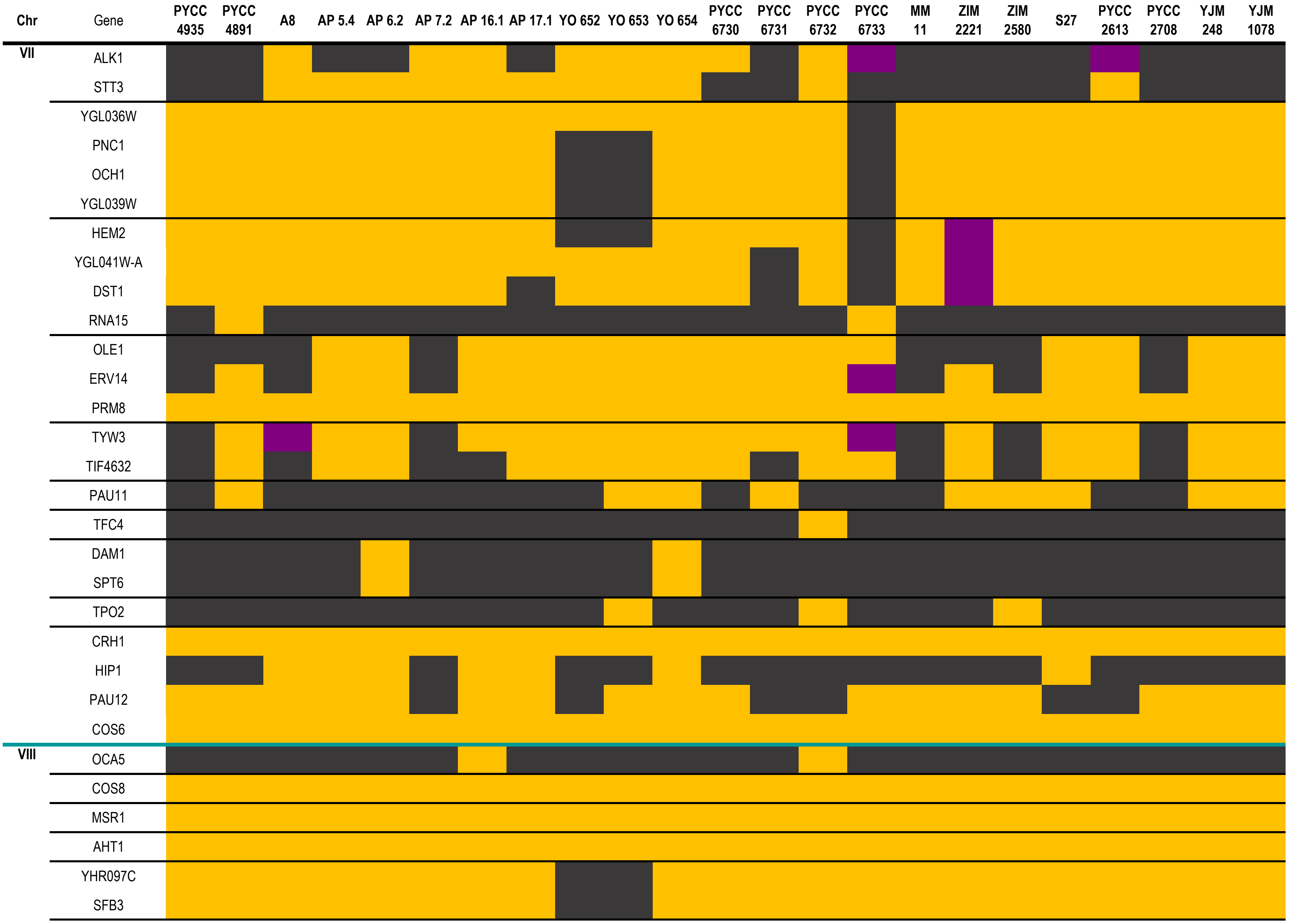

Chr
Gene
PYCC 4935
PYCC 4891
A8
AP 5.4
AP 6.2
AP 7.2
AP 16.1
AP 17.1
YO 652
YO 653
YO 654
PYCC 6730
PYCC 6731
PYCC 6732
PYCC 6733
MM 11
ZIM 2221
ZIM 2580
S27
PYCC 2613
PYCC 2708
YJM 248
YJM 1078
VII
ALK1
STT3
YGL036W
PNC1
OCH1
YGL039W
HEM2
YGL041W-A
DST1
RNA15
OLE1
ERV14
PRM8
TYW3
TIF4632
PAU11
TFC4
DAM1
SPT6
TPO2
CRH1
HIP1
PAU12
COS6
VIII
OCA5
COS8
MSR1
AHT1
YHR097C
SFB3

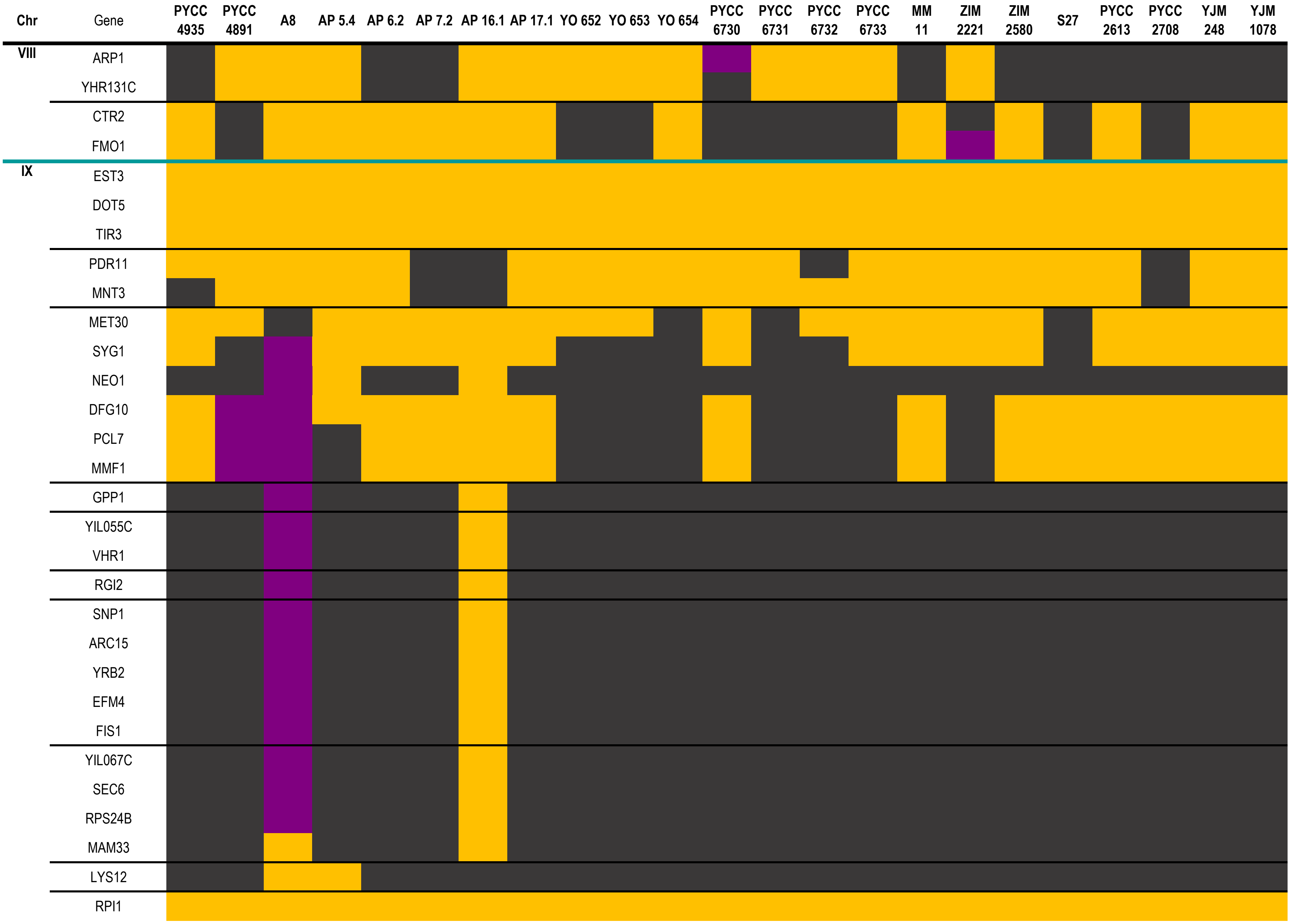

Chr
Gene
PYCC 4935
PYCC 4891
A8
AP 5.4
AP 6.2
AP 7.2
AP 16.1
AP 17.1
YO 652
YO 653
YO 654
PYCC 6730
PYCC 6731
PYCC 6732
PYCC 6733
MM 11
ZIM 2221
ZIM 2580
S27
PYCC 2613
PYCC 2708
YJM 248
YJM 1078
VIII
ARP1
YHR131C
CTR2
FMO1
IX
EST3
DOT5
TIR3
PDR11
MNT3
MET30
SYG1
NEO1
DFG10
PCL7
MMF1
GPP1
YIL055C
VHR1
RGI2
SNP1
ARC15
YRB2
EFM4
FIS1
YIL067C
SEC6
RPS24B
MAM33
LYS12
RPI1

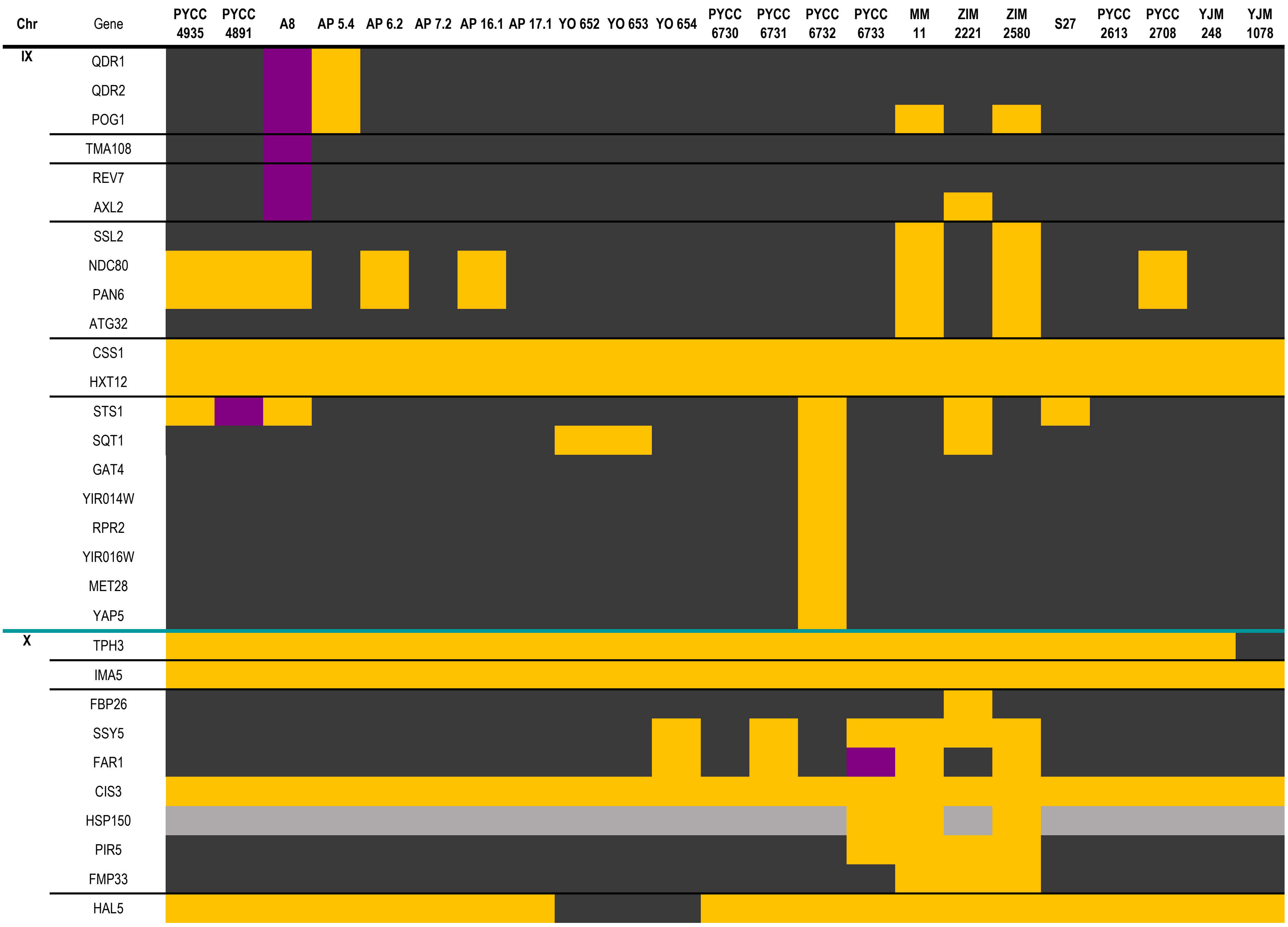
Chr
Gene
PYCC 4935
PYCC 4891
A8
AP 5.4
AP 6.2
AP 7.2
AP 16.1
AP 17.1
YO 652
YO 653
YO 654
PYCC 6730
PYCC 6731
PYCC 6732
PYCC 6733
MM 11
ZIM 2221
ZIM 2580
S27
PYCC 2613
PYCC 2708
YJM 248
YJM 1078
IX
QDR1
QDR2
POG1
TMA108
REV7
AXL2
SSL2
NDC80
PAN6
ATG32
CSS1
HXT12
STS1
SQT1
GAT4
YIR014W
RPR2
YIR016W
MET28
YAP5
X
TPH3
IMA5
FBP26
SSY5
FAR1
CIS3
HSP150
PIR5
FMP33
HAL5

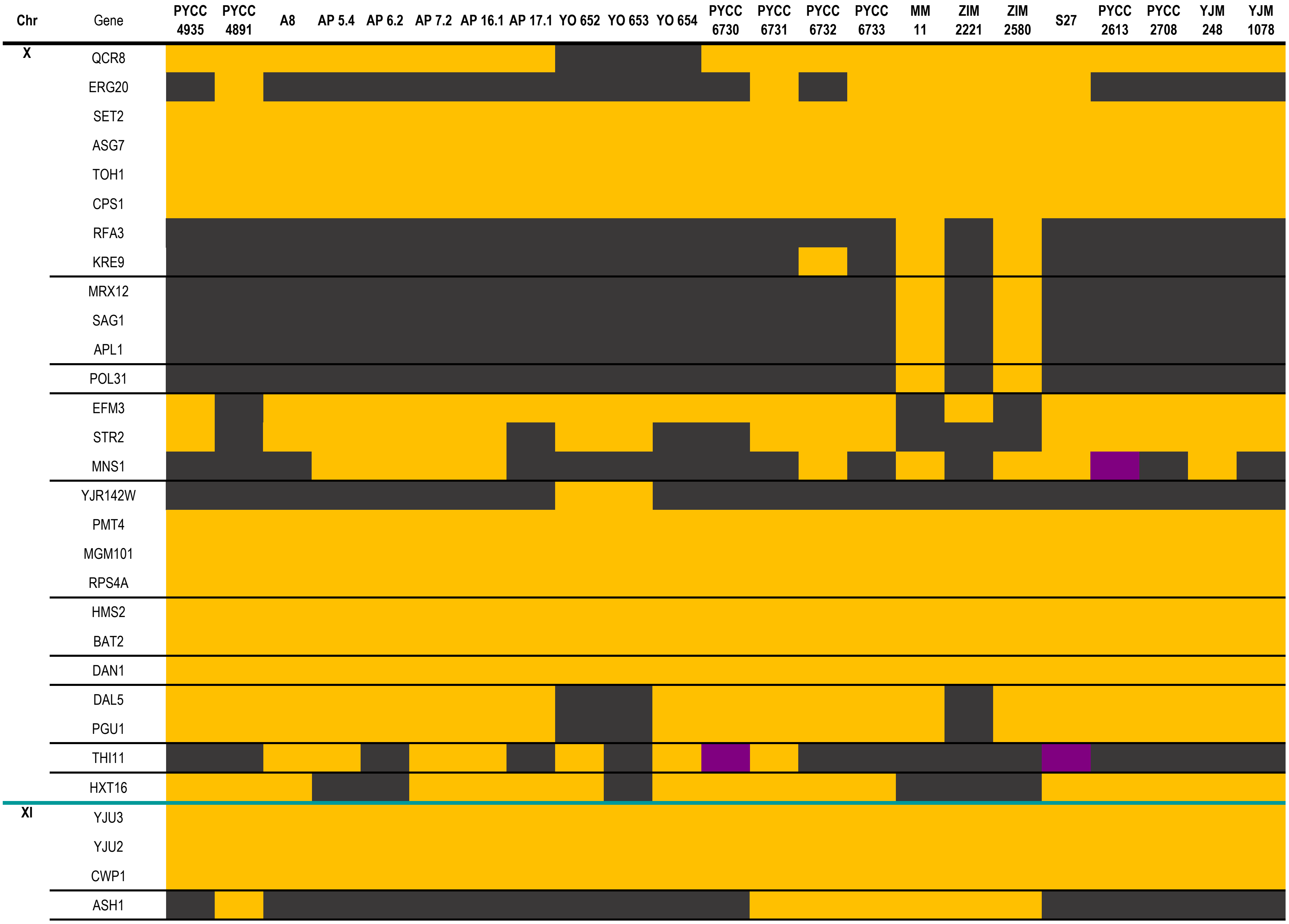
Chr
Gene
PYCC 4935
PYCC 4891
A8
AP 5.4
AP 6.2
AP 7.2
AP 16.1
AP 17.1
YO 652
YO 653
YO 654
PYCC 6730
PYCC 6731
PYCC 6732
PYCC 6733
MM 11
ZIM 2221
ZIM 2580
S27
PYCC 2613
PYCC 2708
YJM 248
YJM 1078
X
QCR8
ERG20
SET2
ASG7
TOH1
CPS1
RFA3
KRE9
MRX12
SAG1
APL1
POL31
EFM3
STR2
MNS1
YJR142W
PMT4
MGM101
RPS4A
HMS2
BAT2
DAN1
DAL5
PGU1
THI11
HXT16
XI
YJU3
YJU2
CWP1
ASH1

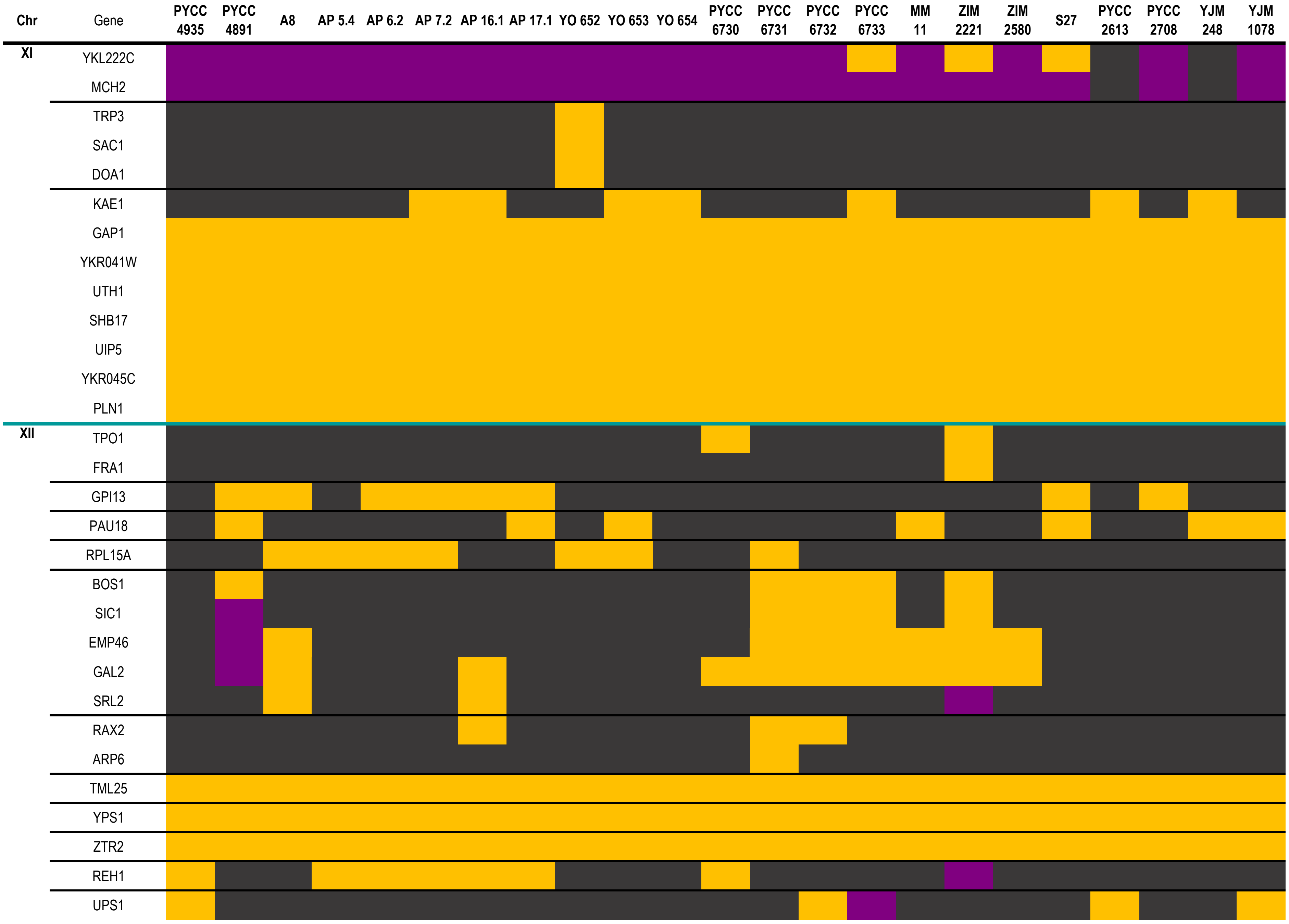

Chr
Gene
PYCC 4935
PYCC 4891
A8
AP 5.4
AP 6.2
AP 7.2
AP 16.1
AP 17.1
YO 652
YO 653
YO 654
PYCC 6730
PYCC 6731
PYCC 6732
PYCC 6733
MM 11
ZIM 2221
ZIM 2580
S27
PYCC 2613
PYCC 2708
YJM 248
YJM 1078
XI
YKL222C
MCH2
TRP3
SAC1
DOA1
KAE1
GAP1
YKR041W
UTH1
SHB17
UIP5
YKR045C
PLN1
XII
TPO1
FRA1
GPI13
PAU18
RPL15A
BOS1
SIC1
EMP46
GAL2
SRL2
RAX2
ARP6
TML25
YPS1
ZTR2
REH1
UPS1

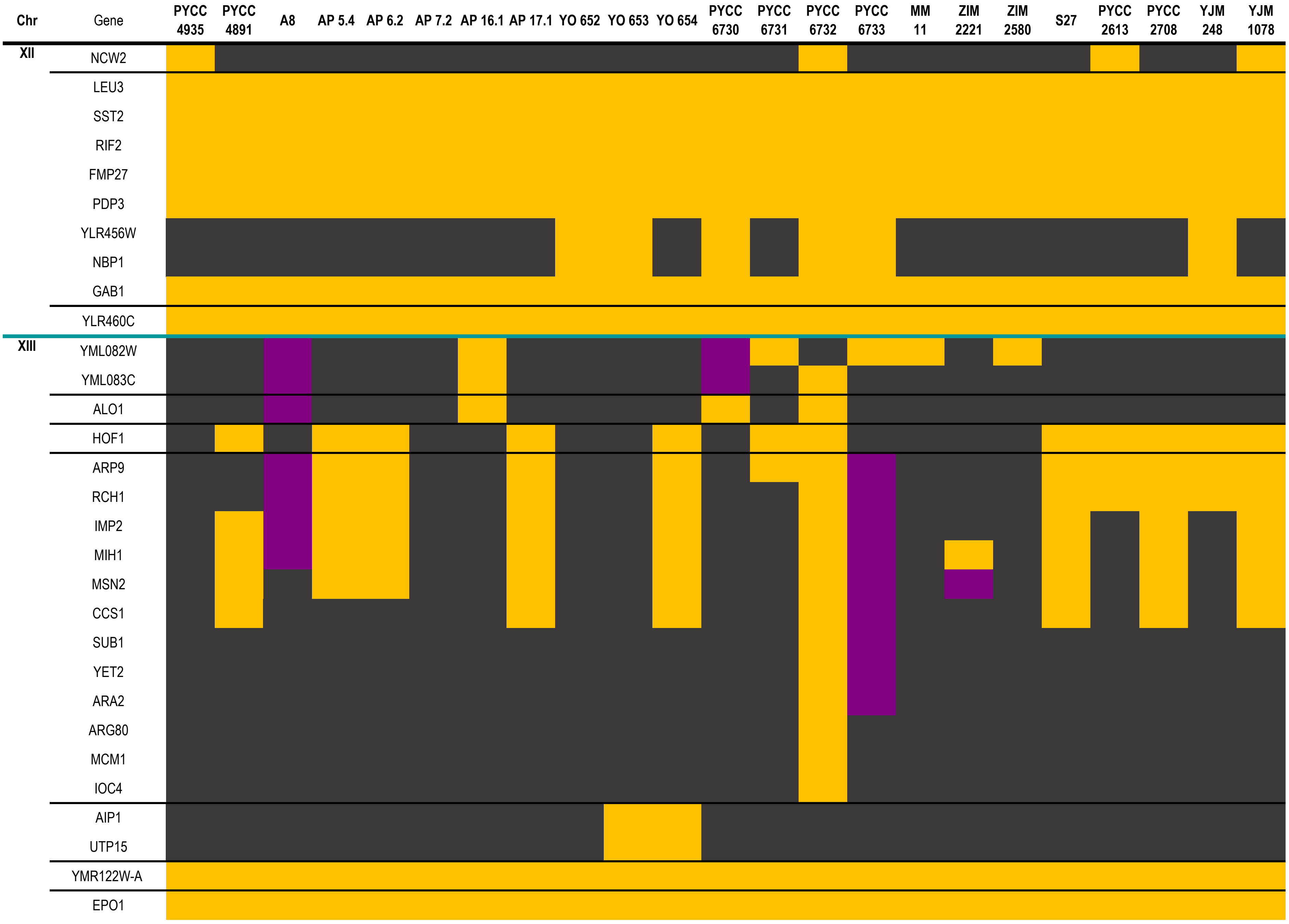
Chr
Gene
PYCC 4935
PYCC 4891
A8
AP 5.4
AP 6.2
AP 7.2
AP 16.1
AP 17.1
YO 652
YO 653
YO 654
PYCC 6730
PYCC 6731
PYCC 6732
PYCC 6733
MM 11
ZIM 2221
ZIM 2580
S27
PYCC 2613
PYCC 2708
YJM 248
YJM 1078
XII
NCW2
LEU3
SST2
RIF2
FMP27
PDP3
YLR456W
NBP1
GAB1
YLR460C
XIII
YML082W
YML083C
ALO1
HOF1
ARP9
RCH1
IMP2
MIH1
MSN2
CCS1
SUB1
YET2
ARA2
ARG80
MCM1
IOC4
AIP1
UTP15
YMR122W-A
EPO1

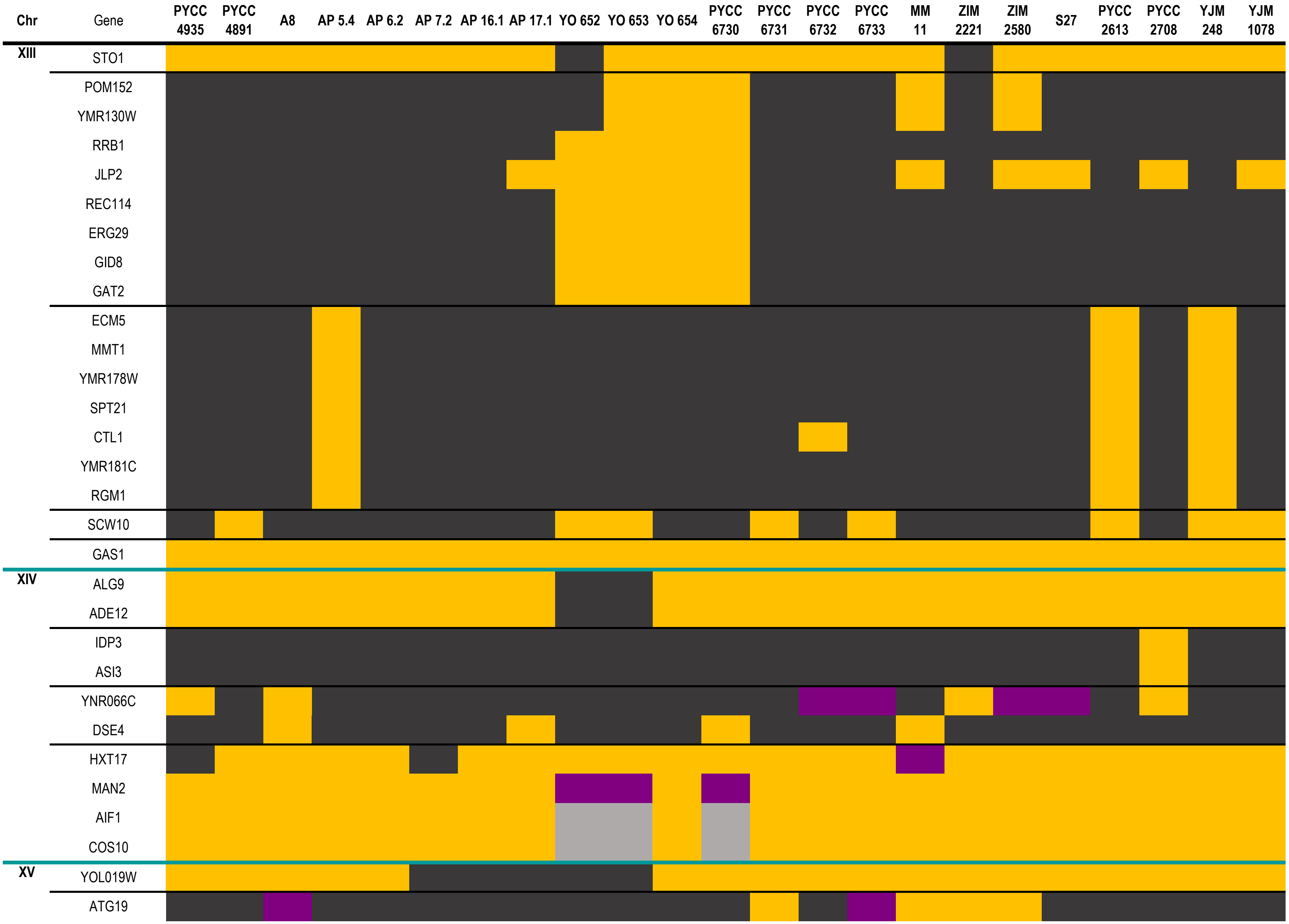
Chr
Gene
PYCC 4935
PYCC 4891
A8
AP 5.4
AP 6.2
AP 7.2
AP 16.1
AP 17.1
YO 652
YO 653
YO 654
PYCC 6730
PYCC 6731
PYCC 6732
PYCC 6733
MM 11
ZIM 2221
ZIM 2580
S27
PYCC 2613
PYCC 2708
YJM 248
YJM 1078
XIII
STO1
POM152
YMR130W
RRB1
JLP2
REC114
ERG29
GID8
GAT2
ECM5
MMT1
YMR178W
SPT21
CTL1
YMR181C
RGM1
SCW10
GAS1
XIV
ALG9
ADE12
IDP3
ASI3
YNR066C
DSE4
HXT17
MAN2
AIF1
COS10
XV
YOL019W
ATG19

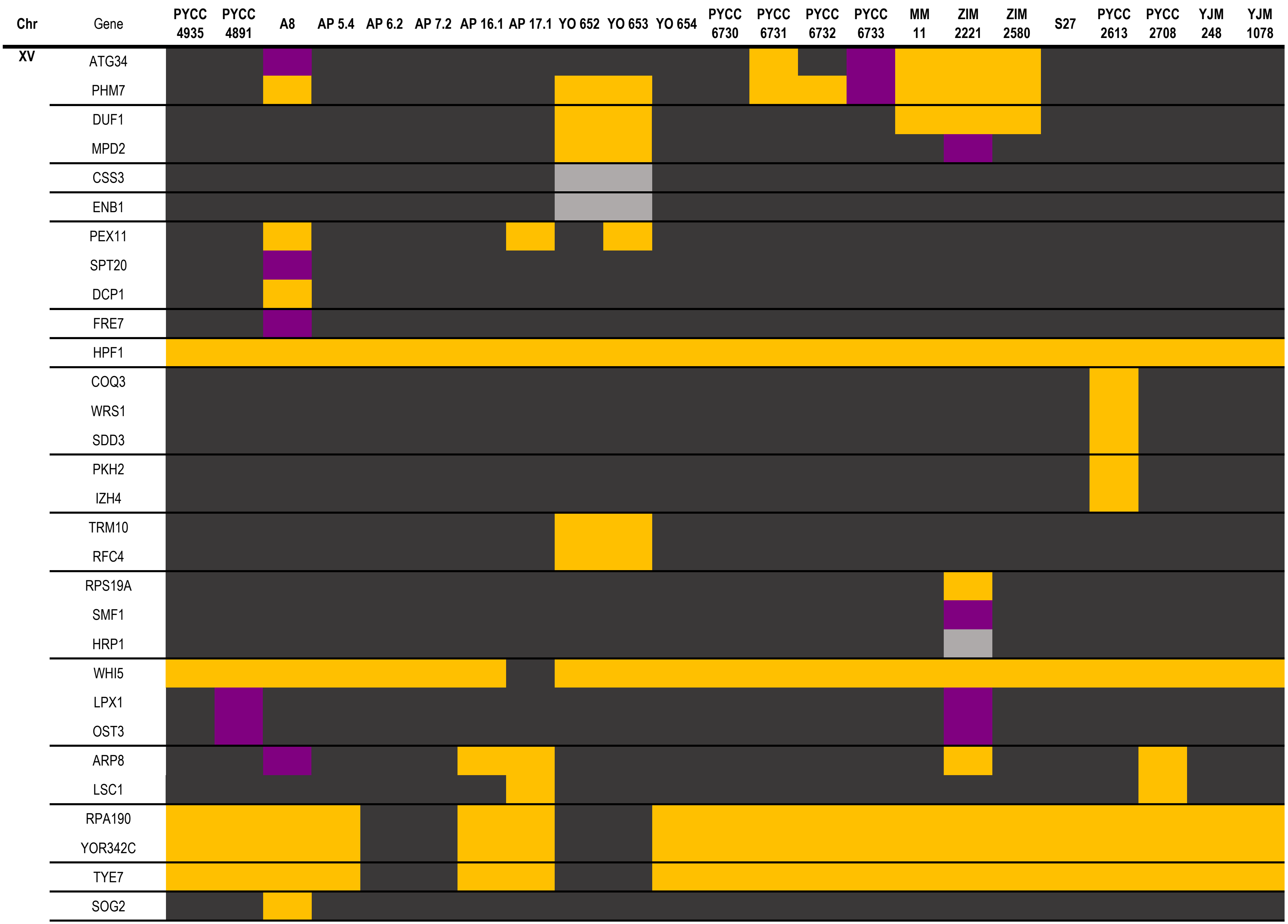
Chr
Gene
PYCC 4935
PYCC 4891
A8
AP 5.4
AP 6.2
AP 7.2
AP 16.1
AP 17.1
YO 652
YO 653
YO 654
PYCC 6730
PYCC 6731
PYCC 6732
PYCC 6733
MM 11
ZIM 2221
ZIM 2580
S27
PYCC 2613
PYCC 2708
YJM 248
YJM 1078
XV
ATG34
PHM7
DUF1
MPD2
CSS3
ENB1
PEX11
SPT20
DCP1
FRE7
HPF1
COQ3
WRS1
SDD3
PKH2
IZH4
TRM10
RFC4
RPS19A
SMF1
HRP1
WHI5
LPX1
OST3
ARP8
LSC1
RPA190
YOR342C
TYE7
SOG2

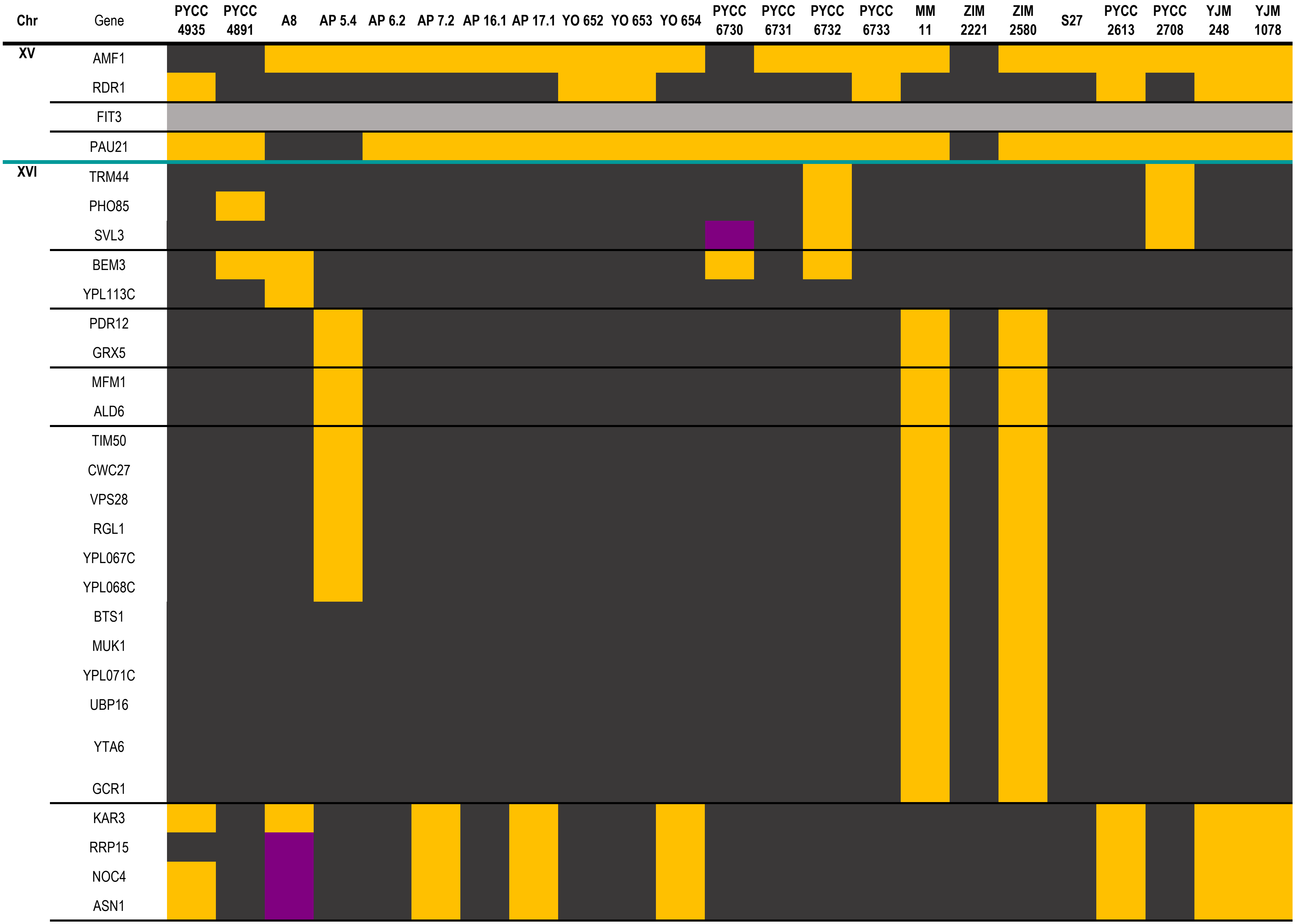
Chr
Gene
PYCC 4935
PYCC 4891
A8
AP 5.4
AP 6.2
AP 7.2
AP 16.1
AP 17.1
YO 652
YO 653
YO 654
PYCC 6730
PYCC 6731
PYCC 6732
PYCC 6733
MM 11
ZIM 2221
ZIM 2580
S27
PYCC 2613
PYCC 2708
YJM 248
YJM 1078
XV
AMF1
RDR1
FIT3
PAU21
XVI
TRM44
PHO85
SVL3
BEM3
YPL113C
PDR12
GRX5
MFM1
ALD6
TIM50
CWC27
VPS28
RGL1
YPL067C
YPL068C
BTS1
MUK1
YPL071C
UBP16
YTA6
GCR1
KAR3
RRP15
NOC4
ASN1

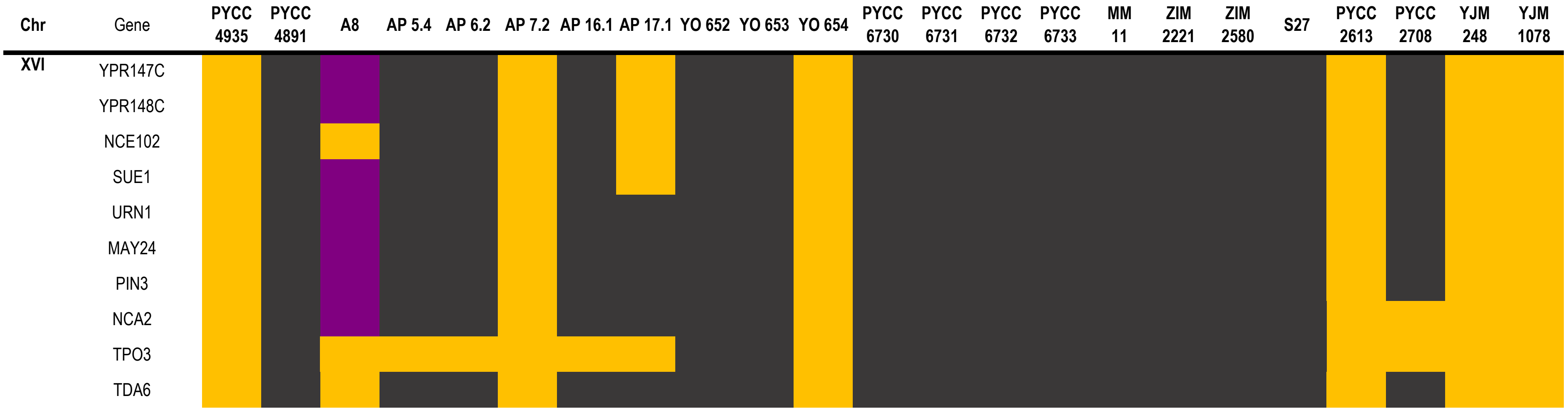

	PYCC 4935	PYCC 4891	A8	AP 5.4	AP 6.2	AP 7.2	AP 16.1	AP 17.1	YO 652	YO 653	YO 654	PYCC 6730	PYCC 6731	PYCC 6732	PYCC 6733	MM 11	ZIM 2221	ZIM 2580	S27	PYCC 2613	PYCC 2708	YJM 248	YJM 1078
***S. paradoxus* genes**	250	254	314	288	246	237	249	275	193	216	282	227	241	271	259	269	269	270	236	268	250	259	244

Shared *S. paradoxus* genes **103**

Blue horizantal line separate chromosomes

Black horizontal line separate gene blocks
